# Nonoptimal component placement of the human connectome supports variable brain dynamics

**DOI:** 10.1162/netn_a_00282

**Published:** 2023-01-01

**Authors:** Christopher James Hayward, Siyu Huo, Xue Chen, Marcus Kaiser

**Affiliations:** Leeds Institute for Data Analytics, University of Leeds, Leeds, United Kingdom; Leeds Institute of Cardiovascular and Metabolic Medicine, University of Leeds, Leeds, United Kingdom; State Key Laboratory of Precision Spectroscopy and School of Physics and Electronic Science, East China Normal University, P. R. China; Institute for Digital Medicine and Computer-Assisted Surgery, Qingdao University, Qingdao, China; Shandong Provincial Key Laboratory of Digital Medicine and Computer-Assisted Surgery, Affiliated Hospital of Qingdao University, Qingdao, China; Shandong College Collaborative Innovation Center of Digital Medicine in Clinical Treatment and Nutrition Health, Qingdao, China; NIHR Nottingham Biomedical Research Centre, School of Medicine, University of Nottingham, United Kingdom; Rui Jin Hospital, Shanghai Jiao Tong University, Shanghai, China

**Keywords:** Component placement optimization, Spatial networks, Wiring minimization, Structural connectivity, Macroconnectome, Metastability, Cognition

## Abstract

Neural systems are shaped by multiple constraints, balancing region communication with the cost of establishing and maintaining physical connections. It has been suggested that the lengths of neural projections be minimized, reducing their spatial and metabolic impact on the organism. However, long-range connections are prevalent in the connectomes across various species, and thus, rather than rewiring connections to reduce length, an alternative theory proposes that the brain minimizes total wiring length through a suitable positioning of regions, termed *component placement optimization*. Previous studies in nonhuman primates have refuted this idea by identifying a nonoptimal component placement, where a spatial rearrangement of brain regions in silico leads to a reduced total wiring length. Here, for the first time in humans, we test for component placement optimization. We show a nonoptimal component placement for all subjects in our sample from the Human Connectome Project (*N* = 280; aged 22–30 years; 138 females), suggesting the presence of constraints—such as the reduction of processing steps between regions—that compete with the elevated spatial and metabolic costs. Additionally, by simulating communication between brain regions, we argue that this suboptimal component placement supports dynamics that benefit cognition.

## Author Summary

The anatomical organization of the brain is shaped by competing constraints for improving brain function while reducing the costs for connectome wiring. Concerning this trade-off, we find that regions within the human brain are not positioned to minimize the total length of their connections. This nonoptimal organization is mainly attributed to frontal and occipital/parietal lobes, with connections between them acting as shortcuts allowing distant brain areas to communicate. By using a model of brain activity, we argue that this suboptimal spatial arrangement of the connectome promotes fluctuations in brain activity, enabling the brain to undertake flexible behavioral responses. Altogether, this highlights that brain structure, while spatially suboptimal, may offer dynamic advantages that support effective cognition.

## INTRODUCTION

The brain consists of numerous spatially distributed, functionally distinct regions. The map of structural connections between brain regions—the human connectome (Sporns, Tononi, & Kötter, 2005)—encompasses long-range links between distant regions, supporting integration, and short-range links mainly within network modules supporting local processing (Watts & Strogatz, 1998). Such an arrangement reduces both the transmission time in terms of the number of intermediate regions on pathways and the total wiring length of the network (Bassett & Bullmore, 2006; Bullmore & Sporns, 2009; Hagmann et al., 2008; Hilgetag & Kaiser, 2004; Seguin, van den Heuvel, & Zalesky, 2018; Sporns, Chialvo, Kaiser, & Hilgetag, 2004), an organization thought to facilitate effective cognition (Cohen & D’Esposito, 2016; Sporns, 2013). Brain network structure and dynamics are strongly linked with different hierarchical organization (Huo, Zou, Kaiser, & Liu, 2022) or different topologies (Muldoon et al., 2016) leading to a wide variability in oscillation and synchronization patterns. However, further research is needed on the interaction between spatial and topological features of the brain, and the influence of spatial features on neural communication.

Concerning the network’s spatial organization, there is a trade-off between the physical and metabolic costs of long-range connections and their benefits for distribution, robustness after network perturbations, and modular/segregated processing (Bullmore & Sporns, 2012). Indeed, there are more long-distance connections than would be expected in the case where wiring is strictly minimized. Thus, rather than rewiring projections to minimize their lengths, an alternative perspective is that the brain minimizes wiring lengths through a suitable spatial arrangement of brain regions, termed *component placement optimization* (CPO). Under such conditions, any other arrangement of the spatial position of brain regions would always result in a longer total wiring length. In other words, it would be impossible to reduce the total wiring length by swapping regions (Cherniak, 1994; Cherniak, Mokhtarzada, Rodriguez-Esteban, & Changizi, 2004). This idea, however, has been refuted in the case of *Caenorhabditis elegans* (a nematode 1 mm in length) and the rhesus monkey (Kaiser & Hilgetag, 2006).

Here, we test for component placement optimization for the first time in humans, and offer a novel insight into the relationship between the spatial arrangement of connectome regions and neural dynamics. Using the method described in Kaiser and Hilgetag (2006), we swap the positions of brain regions in silico in an attempt to identify arrangements that reduce the total wiring length. Using the structural connectivity of 280 subjects from the Human Connectome Project (Van Essen et al., 2012) (aged 22–30 years; 138 females), we find that the human connectome, as previously found for the rhesus monkey (Kaiser & Hilgetag, 2006), shows nonoptimal component placement—a reduction in total wiring length is possible by rearranging regions while maintaining the connectome topology. Some regions affected the change in total wiring length more than others; regions in the frontal, occipital, and parietal lobes could be relocated to significantly reduce the length of their connections to other regions, while on the other hand, connections from subcortical regions and the insular cortex showed fewer changes during optimization. In addition to providing direct communication between distant regions, we argue that this suboptimal arrangement supports dynamics that are associated with optimal network performance (Friston, 1997). By modeling the effects of suboptimal arrangements on neural activity within the brain, we observe changes in synchronization between regions—a suggested mechanism of communication (Fries, 2015; von der Malsburg, 1995)—highlighting a novel relationship between region placement and dynamics. Altogether, this suggests that the spatial organization of the human connectome is shaped by multiple constraints: balancing the spatial and metabolic costs of white matter connections between regions, while ensuring the emergence of metastable dynamics that are necessary for effective cognition (Deco, Rolls, & Romo, 2009).

## RESULTS

### Reduction in Total Wiring Length by Swapping Brain Regions

Each connectome consisted of 82 regions—34 cortical and 7 subcortical regions per hemisphere (Desikan et al., 2006)—with 1,012 ± 67 connections on average (edge density equal to 0.31 ± 0.02), and an average normalized straight-line wiring length of 0.41 ± 0.01 (divided by the maximum Euclidean distance across each connectome). Table 1 displays the differences in connectome structure between age groups and sex. Compared with males, females had a greater edge density (*P* < 0.01, permutation test) and longer normalized wiring (*P* < 0.01).

**Table 1.** Connectome features across age groups and sex

	Age: 22–25 years (*N* = 91)	Age: 26–30 years (*N* = 189)	*P*_age_
Edge density	0.308 ± 0.021	0.304 ± 0.02	0.10
Mean wiring length	0.409 ± 0.015	0.408 ± 0.013	0.61
Relative wiring length	0.451 ± 0.034	0.449 ± 0.03	0.29*
	Males (*N* = 142)	Females (*N* = 138)	*P*_sex_
Edge density	0.302 ± 0.02	0.308 ± 0.02	0.009
Mean wiring length	0.406 ± 0.013	0.411 ± 0.014	0.002
Relative wiring length	0.443 ± 0.033	0.456 ± 0.029	0.52*

*Note*. *P* values are calculated using a two-tailed permutation test (one million permutations). The mean wiring length uses the Euclidean distance, normalized using the maximum Euclidean distance across all regions (= 1).

* Because of the interaction between relative wiring length and edge density (Supporting Information Figure S1), the relative wiring length *P* values are corrected using edge density. Values are mean ± *SD*.

**Figure 1.**
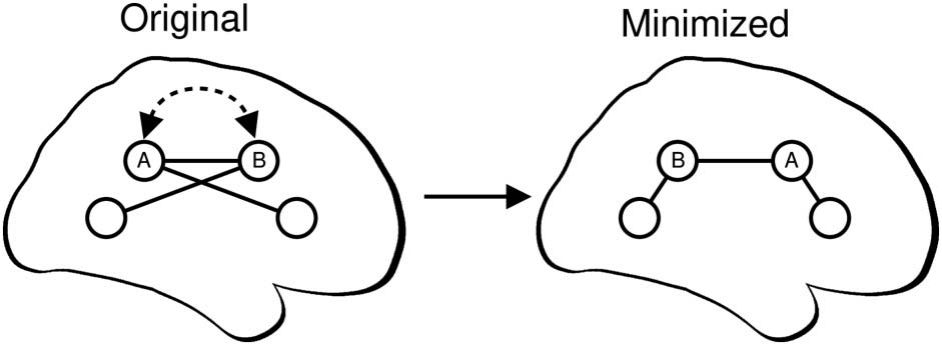
Overview of component placement optimization procedure. Brain regions within each connectome (circles) are swapped to reduce the total wiring length (the sum of the length of all edges—straight black lines). Here, regions A and B are swapped (curved, dashed arrow), resulting in the “minimized” connectome. The network topology does not change during this process. For each subject, we identify a minimized arrangement by sampling from ≈4.8 × 10^122^ possible arrangements of 82 regions (see the Materials and Methods section).

**Figure 2.**
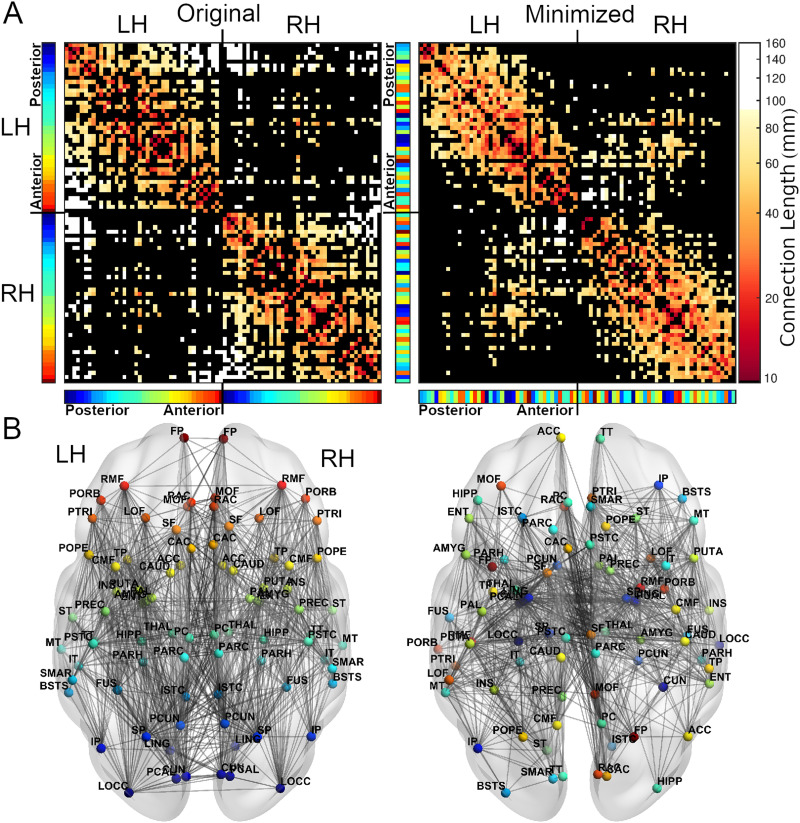
For an example subject, the adjacency matrices (A) and axial views (B) of the original (left) and minimized (right) arrangement of regions. In the minimized arrangement, the spatial positions of regions are swapped to reduce the total wiring length. Regions are colored based on their anterior-posterior position in the original arrangement, displayed in the color bars next to the adjacency matrices (red = anterior, blue = posterior). The topology of the connectome is preserved in both arrangements, but the positions of regions often differ. White connections in the adjacency matrices are those with lengths greater than 80% of connections in the original arrangement. See Supporting Information Table S1 for full region names. L/RH = left/right hemisphere.

**Figure 3.**
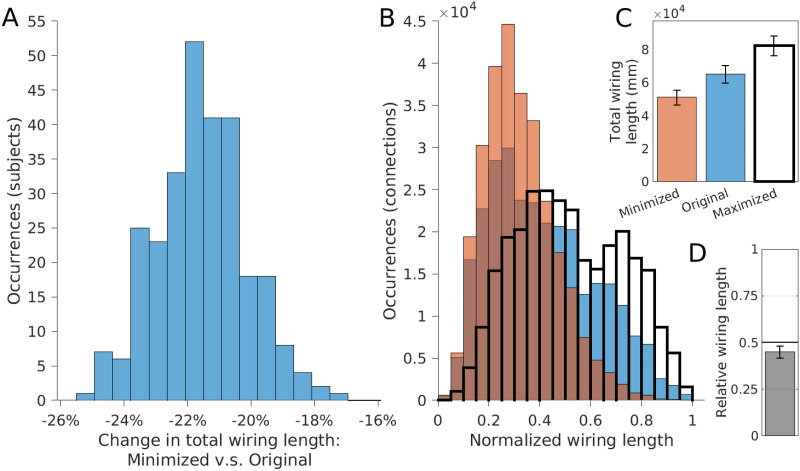
For all 280 subjects: changes in wiring length distribution between original, minimized, and maximum arrangements. (A) The distribution of wiring length changes, from original to minimized arrangements (mean reduction of 21.6 ± 1.4%. (B) The distribution of wiring lengths for original (blue), minimized (orange), and maximized (transparent, bold) spatial arrangements. (C) The mean wiring lengths for minimized, original, and maximized arrangements. (D) The mean relative wiring lengths—original lengths relative to the minimized and maximized wiring lengths (mean 0.45 ± 0.03) (error bars = 1 *SD*).

To assess whether the human brain displays component placement optimization, we iteratively swapped the positions of random pairs of regions for each subject individually, with the goal of reducing the total wiring length for each subject as much as possible (Figure 1; see the Materials and Methods section). Alongside the “original” arrangement, this process converged to produce a “minimized” spatial arrangement for each subject (Figure 2; see Supporting Information Table S1 for full names of each region). As a result, we were able to reduce the total wiring length for all 280 subjects, with an average reduction in wiring length of 21.6 ± 1.4% (Figure 3A); that is, the original arrangement was spatially suboptimal for all subjects. Minimized arrangements displayed fewer long-distance connections (Figure 3B). While searching for a reduced wiring length would be the best-case scenario of saving wiring, we also looked at the worst-case scenario of increasing the total wiring length as much as possible. Using such “maximized” networks, we calculated the relative total wiring length of the original network where a value of 0 represents the best case (minimal wiring length) and a value of 1 represents the worst case (maximal wiring length). The mean relative wiring length equated to 0.45 ± 0.03, with 265 subjects (95%) having a value under 0.5 (ranging from 0.33 to 0.54), indicating the original spatial arrangement of regions tended to align more closely with the best-case scenario of minimizing the wiring length (Figure 3D). The relative wiring length was reduced for connectomes with fewer connections (Supporting Information Figure S1), but did not differ between sex or age groups, or between isolated hemispheres (Supporting Information Figure S2).

**Figure 4.**
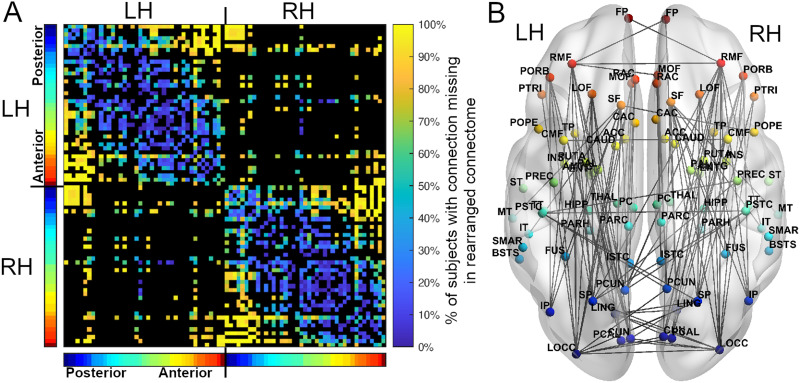
Across all 280 subjects, the connections that disappeared after rearranging regions. (A) For the connections that were present in at least 50% of subjects, an adjacency matrix depicting the percentage of subjects that had a connection between two positions in the original arrangement but did not have the same connection between the same two positions in the minimized arrangement. Black regions represent connections appearing in fewer than 50% of subjects. (B) Of the connections shown in A, an axial view of the connections that disappeared in more than 90% of subjects. Note that connection lengths between hemispheres are scaled to pass through the center-of-mass of the connectome, and are therefore longer than shown here (see the Materials and Methods section). Regions are colored based on their anterior-posterior position in the original arrangement, displayed in the color bars next to the adjacency matrix (red = anterior, blue = posterior). L/RH = left/right hemisphere.

Regarding the connections that were most often missing between spatial positions in the minimized arrangements, and thus the most suboptimal connections in the connectome (those that no longer existed between spatial positions in the minimized connectomes), missing connections were often inter- and intrahemispheric between anterior and posterior positions (Figure 4, Supporting Information Table S2). Spatial positions in the occipital and frontal lobes saw the greatest number of missing connections in the minimized arrangements, losing on average 83 ± 24% and 74 ± 30% of their original projections, while subcortical positions lost the fewest (48 ± 34%). Longer connections disappeared more frequently (Supporting Information Figure S3), and interhemispheric versus intrahemispheric connections were more likely to disappear, with a mean percentage of disappearance of 77 ± 18% and 29 ± 25% respectively.

Our approach of swapping regions assumed that any region could be swapped with any other, irrespective of volume differences. In reality, swapping regions of different volumes will alter the positions of neighboring regions, affecting the overall reduction in wiring length. We therefore checked to see whether reductions in wiring length were still possible when only regions with similar volume were allowed to swap positions (Supporting Information Figure S4). Even when two regions can only swap positions if their volumes differ by no more than ±5%, a total wiring length reduction still occurred for all subjects, reducing by 3.4 ± 1.2% compared with 21.6 ± 1.4% when swapping without volume constraints. Moreover, reductions in wiring length were still possible after removing weak connections, that is, those with few streamlines (Supporting Information Figure S5). In fact, as more connections were removed and the rearrangement procedure became less constrained, a greater reduction in total wiring length was observed.

### Changes in Wiring Length for Individual Regions

**Figure 5.**
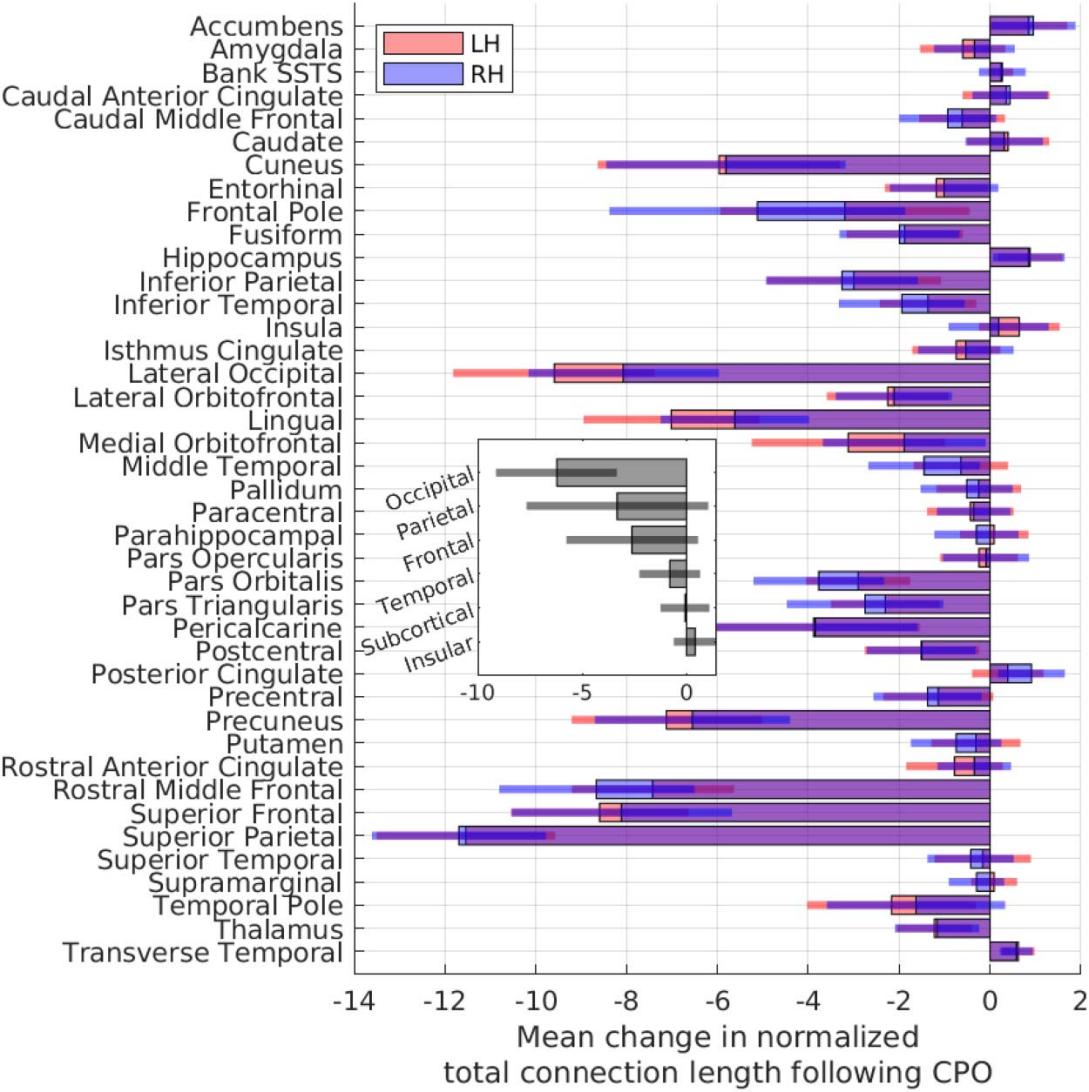
For all 280 subjects, the change in total connection length per region (sum of lengths of all connections attached to a region) after repositioning into the minimized arrangements (i.e., following component placement optimization: CPO). The lengths of connections are normalized using the maximum Euclidean distance [= 1] between all regions, per subject. Inset: mean per lobe. Error bars = 1 *SD*. L/RH = left/right hemisphere.

To further our analysis of the minimized arrangements, we explored the extent to which individual brain regions contribute towards reducing the wiring length in the minimized arrangements. Comparing the wiring length of each region’s connections before and after rearrangement, we found that wiring lengths reduced for almost all regions in the minimized arrangements, with changes broadly consistent across hemispheres (Figure 5). Regions in the extreme anterior and posterior areas experienced the greatest reduction in wiring length, particularly the lateral occipital and superior parietal regions. The occipital lobe experienced the greatest reduction in wiring length, while insular and subcortical regions contributed the least towards changes in the wiring length. Interestingly, connection lengths increased for some regions, in particular the nucleus accumbens, hippocampus, and transverse temporal areas. These regions often moved to positions that increased the total wiring length, likely replaced by regions that made more efficient use of the spatial position. Regions with longer connections, as well as those on the periphery of the connectome, experienced a greater reduction in wiring length following rearrangement (Supporting Information Figure S6).

### Functional Implications of Suboptimal Arrangements

**Figure 6.**
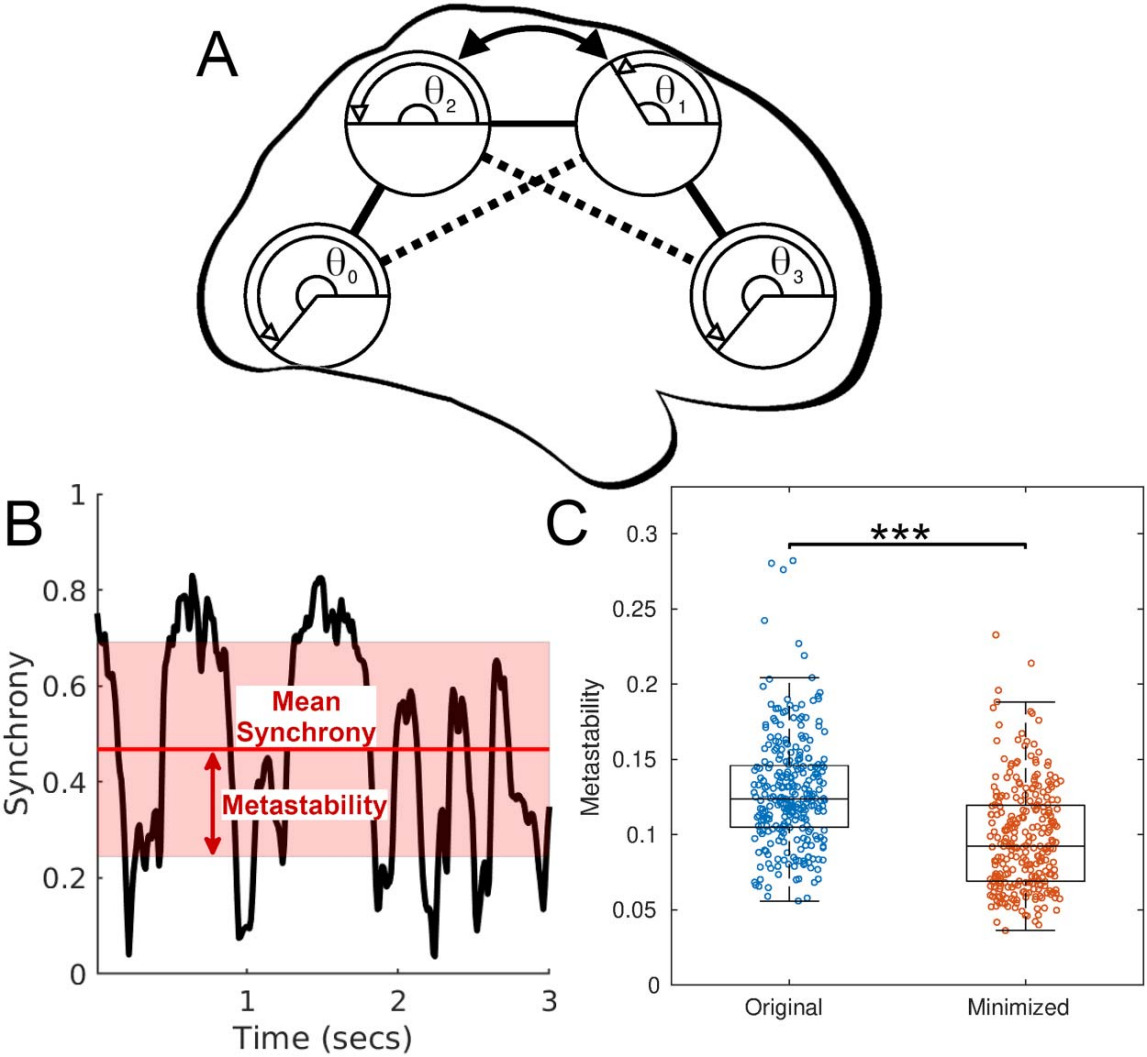
Simulating changes in neural activity for original and minimized connectomes. (A) A simplified overview of the model setup: The dynamics of brain regions are modeled using Kuramoto oscillators (phase *θ*), connected by edges, displayed here in a minimized arrangement, sagittal view (the original arrangement is obtained by swapping regions 1 and 2—indicated by the arrow and dashed lines). (B) A snippet of example activity showing the change in synchrony over all 82 regions (black line) and the corresponding mean synchrony (red line) and metastability (variance of synchrony, shaded red). (C) Across all subjects, metastability for the original and minimized arrangements at 60 Hz (*** = *P* < 0.001, Wilcoxon signed-rank; Cohen’s *d* = 0.81). Seventy-eight percent of subjects displayed reduced metastability for their minimized arrangements.

Having identified that optimal positioning of regions with respect to wiring length does not occur in the human connectome, we lastly explored the functional implications for suboptimal arrangements by hypothesizing that such arrangements act as a structural foundation supporting beneficial neural dynamics. To assess changes in dynamics for each connectome, we replicated neural dynamics in silico using models composed of 82 Kuramoto coupled oscillators (Kuramoto, 1975). Kuramoto oscillators are commonly used to simulate interactions between neural populations (Cabral, Hugues, Sporns, & Deco, 2011; Hellyer, Scott, Shanahan, Sharp, & Leech, 2015; Váša et al., 2015). Here, each oscillator represents the neural dynamics of an individual brain region, connected to one another according to the connectivity defined by each subject’s connectome (Figure 6A). Between all regions, we measured mean synchronization (correlated oscillatory neural activation across all regions) as well as metastability (variance in mean synchronization across the duration of the simulation—Figure 6B). Synchronization is suggested to underpin communication between regions (Fries, 2015), while high metastability reflects regular transitions between segregation and integration of neural populations, dynamics necessary for flexible coordination between neural processes (Deco, Rolls, & Romo, 2009; Friston, 1997) and task switching (Hellyer et al., 2015). We measured synchronization as the difference in phase between all oscillators within each connectome; two oscillators are synchronized (synchronization equals 1) when their phase difference is 0. Conduction velocities (speed of propagation of action potentials between regions) and coupling strengths (the weight of influence that each oscillator has on their connected oscillators) were selected by comparing the model’s activity with functional connectivity obtained from empirical resting-state fMRI data per subject (see the Supporting Information). We used a 30 × 30 parameter space to select the parameters that best matched the empirical data (velocities ranging from 1 to 30 m/s, and strengths from 1 to 30). We repeated our analyses for three gamma-band frequencies—40 Hz, 60 Hz, and 80 Hz—corresponding to the natural frequency of oscillation for each region.

Across all subjects, for 60 Hz, the fMRI-validated mean conduction velocities and coupling strengths were 12.9 ± 3.9 m/s and 13.5 ± 4.3, respectively (see Supporting Information Figures S7 and S9 for 40 Hz and 80 Hz). At 60 Hz, the mean values of synchrony and metastability were 0.51 ± 0.04 and 0.13 ± 0.04, respectively (for metastability, this equated to 59 ± 15% of the maximum metastability achieved over the parameter space). Because simulated activity generated from the minimized arrangements cannot be validated against empirical fMRI data, we calculated the corresponding metastability for the minimized arrangements by identifying the velocity/strength parameters, per subject, which gave rise to a level of synchronization comparable to that of the models validated against the original arrangements (Fukushima & Sporns, 2020; Váša et al., 2015) (Supporting Information Figures S7–S9). This approach meant that synchronization was restricted to be as similar as possible between spatial arrangements, enabling a comparison in metastability (see the Supporting Information).

Minimized arrangements displayed significantly reduced metastability compared with the original arrangements—at 60 Hz, metastability was lower for the minimized arrangements for 217 out of the 280 subjects (78%) (*P* < 0.001, Wilcoxon signed-rank; Cohen’s *d* = 0.81; Figure 6C). Metastability also decreased for minimized arrangements at 40 Hz (*P* < 0.001; Cohen’s *d* = 0.76; reduced in 222 subjects [79%]) and 80 Hz (*P* < 0.001; Cohen’s *d* = 0.76; reduced in 220 subjects [79%]) (Supporting Information Figure S10). Metastability reduced on average by 19 ± 28%, 20 ± 29%, and 19 ± 27%, for 40 Hz, 60 Hz, and 80 Hz, respectively. When considering the top 50% of subjects with the closest match with the fMRI data, significant reductions in metastability for the minimized arrangements were observed across all three frequencies (*P* < 0.001, Wilcoxon signed-rank; Cohen’s *d* = 0.84 [40 Hz], 0.93 [60 Hz], 1.02 [80 Hz]), reducing in 81%, 83%, and 83% of subjects for 40 Hz, 60 Hz, and 80 Hz, respectively (Supporting Information Figure S11). Significant overall reductions in metastability still occurred (*P* < 0.001) irrespective of differences in mean synchronization between arrangements (Supporting Information Figure S12).

## DISCUSSION

In this study we explored the extent to which the human brain minimizes wiring length through a suitable spatial arrangement of regions, a theory termed component placement optimization. By analyzing wiring length distributions and rearranging regions within the connectome, we have provided evidence for the existence of a suboptimal arrangement of brain regions with respect to minimizing the total length of projections. In an attempt to identify a reason for why this suboptimal arrangement exists, we found reduced variation in the dynamics produced by the minimized arrangements compared with the original arrangements. The overarching result is that a suboptimal arrangement may offer functional benefits by supporting dynamics that facilitate flexible coordination between neural processes, despite the increased spatial and metabolic costs.

### Component Placement Is Suboptimal in the Human Connectome

Alongside the longer connections and increased connectivity in females, the lack of difference in relative wiring length between sexes implies a similar suboptimal connectivity in both males and females, and where the additional connectivity in females does not affect optimal wiring configurations. Similar levels of optimization were also found in both age groups, demonstrating that suboptimal wiring persists across age, albeit across the limited age range and coarse age categories used in this study.

Our work extends the findings of other studies (Chen, Hall, & Chklovskii, 2006; Kaiser & Hilgetag, 2006) by identifying nonoptimal component placement in the human connectome. Long-distance connections contributed to the nonoptimal wiring in the original placement, often attributed to connections between occipital/parietal and frontal regions. Long-range connections have been observed across a variety of neural systems (Bassett & Bullmore, 2006), but here we find that the spatial arrangement of regions is not organized to minimize these projections, suggesting the presence of constraints that oppose the need to minimize spatial and metabolic costs of connections. A possible benefit of long-range connections is to allow direct connectivity to distant regions, bypassing processing steps, reducing communication delays (Kaiser & Hilgetag, 2006), and supporting integration between regions (Samu, Seth, & Nowotny, 2014).

In the minimized arrangements, the connection lengths decreased for almost all regions, suggesting an overall suboptimal positioning of brain regions. This reduction was dominated by connections emanating from peripheral regions, particularly from the lateral occipital, superior parietal, and rostral middle frontal areas. In particular, the lengths of connections from the occipital lobe could be greatly reduced in the rearranged networks. This included anterior-directed projections, coinciding with the inferior fronto-occipital fasciculus, superior longitudinal fasciculus, and cingulum. The suboptimal placement of the occipital lobe, given its connections, suggests a need for uninterrupted communication with distant regions, despite the increased wiring costs. The importance of maintaining direct connectivity is supported by the quantity of anterior-directed streamlines from the occipital lobe, more than would be expected based on connection length alone (Roberts et al., 2016). From an evolutionary perspective, the occipital lobe versus other lobes is comparatively old and may have to compensate for its position with the use of long-distance connections to regions that show greater changes during evolution such as the frontal lobe (Hill et al., 2010).

### Suboptimal Placement of Regions May Support Variable Brain Dynamics

While reducing the length of connections may reduce the amount of resources needed for connection establishment and maintenance, our dynamical model suggests that a suboptimal arrangement of regions, which includes long-range connectivity—in particular, emanating from frontal and occipital/parietal lobes—offers functional advantages by supporting transitions between synchronized and desynchronized states. Such states correspond to periods of integration and segregation between regions (Fries, 2015), features that are crucial for healthy brain function (Deco, Rolls, & Romo, 2009). Many of the regions with missing connections overlapped with those implicated in task performance (Jung & Haier, 2007), particularly frontal and parietal areas, regions that are prone to disconnection under cost-preserving rewirings of the connectome (Gollo et al., 2018). Metastable dynamics have been attributed to lateral regions of the connectome in other studies (Váša et al., 2015), aligning with not only our finding of the spatial influence on dynamics, but also with the suboptimal placement of regions—those on the periphery of the connectome. Our results suggest that regions on the periphery of the connectome may be critical to maintaining variable brain dynamics.

The perceived effects of long-range connectivity coincides with the metastable dynamics of small-world networks that naturally consist of connections of different lengths (Wildie & Shanahan, 2012). While other studies have highlighted the importance of network features such as clusters and hub nodes in supporting integrated and segregated activity (Sporns, 2013), our results emphasize that spatial features may also contribute towards such dynamics (Fukushima & Sporns, 2020; Seguin et al., 2018), and that minimizing wiring length may be detrimental to healthy brain function.

Similar in nature to pipelining in semiconductor processors where initial and subsequent instructions are processed concurrently (Shen & Lipasti, 2013), a mixture of long- and short-range connections, giving rise to a temporal staggering of processing steps, may promote binding between distant regions (von der Malsburg, 1995). Changes in the distribution of communication delays between regions, caused by swapping regions, may be driving the changes in dynamics (Deco, Jirsa, McIntosh, Sporns, & Kötter, 2009). The level of synchronization enabled by a suboptimal spatial arrangement may support a broader exploration of system states, and a wider repertoire of behavioral responses.

The rearrangement method used in this study is analytic in nature, limited in its level of biological realism. Indeed, regions often moved far from their original position, with a median [Q1, Q3] displacement of 54 mm [37 mm, 71 mm] (Supporting Information Figure S13). Many features that are known to affect wiring length are missing from this analysis, particularly the shape of regions and their connections. In our analysis, we used the Euclidean distance between regions to analyze wiring lengths, whereas real fiber tracts will deviate from a straight line. For our subjects, subsequent analysis revealed that fiber tracts were on average 78 ± 100% longer than their Euclidean approximation. Moreover, conduction speeds will be affected by varying amounts of myelination (Doron & Gazzaniga, 2008; Waxman, 1977), a feature that is not accounted for in our model and one that may influence synchronization (Park & Lefebvre, 2020). Consideration of the volume occupied by myelinated axons may lead to further reductions inspatial costs, caused by a reduced reliance on myelination in the minimized arrangements. Future studies could consider axon diameter and the physical shape of fiber tracts when testing CPO and the effect on metastable dynamics. Nonetheless, by constraining regions to stay within their hemispheres, lengthening interhemispheric connections (Supporting Information Figures S14 and S15), and considering differences across region volumes in a separate analysis, we have attempted make our findings more biologically plausible, but further work is needed to develop a more detailed swapping method that takes into account the shape of regions and the meandering of their connections.

In summary, our results suggest that the arrangement of regions within the human connectome does not minimize the total wiring length. This is the first time that component placement optimization has been performed in the human connectome. In our sample of subjects from the Human Connectome Project, locations of brain regions could be rearranged to reduce the total wiring length; the human connectome allows for specific long-distance connections, in particular fiber tracts connecting frontal and occipital/parietal regions. Using the novel approach of pairing a model of neural oscillations with different spatial arrangements of the connectome, we found that the connections involved in nonoptimal component placement appear to enable bouts of integrated and segregated activity through changes in synchronization between regions. Combined with previous studies (Kaiser & Hilgetag, 2006), our results lend support for a universal law of connectome organization across species, contrasting with suggestions of wiring optimization in neural systems (Cherniak, 1994). Overall, despite the increased wiring cost, a suboptimal arrangement may be beneficial for maintaining activity that supports flexible communication between regions, and subsequently, increased cognitive processing performance (Fries, 2015; von der Malsburg, 1995).

## MATERIALS AND METHODS

### Data

Our study consisted of 280 healthy adults aged between 22 and 30. Structural, diffusion, and functional data were obtained from the Human Connectome Project (Van Essen et al., 2012; HCP; https://www.humanconnectome.org; S1200 release). For structural and diffusion data, we used T1-weighted 3T MRI and diffusion imaging preprocessed data. Preprocessed data included a FreeSurfer parcellation of each subject’s T1 image into 34 cortical and seven subcortical regions (amygdala, caudate, hippocampus, nucleus accumbens, pallidum, putamen, and thalamus) (Fischl et al., 2002) per hemisphere, based on the Desikan atlas (Desikan et al., 2006). This resulted in a parcellation of 82 brain regions per subject. The rs-fMRI BOLD data were obtained from the ICA-FIX denoised dataset (see the Supporting Information).

We assessed wiring length optimization by swapping brain regions and testing whether the total length of all connections—estimated by the sum of the Euclidean distance of connections between regions—could be reduced (Figure 1). The topology of the connectome does not change during this rearranging process, but connections between spatial positions will often differ between arrangements in such a way that reduces the total connection length in the connectome. As in the previous study (Kaiser & Hilgetag, 2006), we identified these arrangements using simulated annealing (see the Supporting Information).

While fiber tracts are approximated as straight lines, this is unrealistic for connections between hemispheres that primarily pass through the corpus callosum. To mimic the presence of interhemispheric constraints and thus improve our approximation of the change in total wiring length, we changed the lengths of interhemispheric connections as if they passed through the center-of-mass of the network (Supporting Information Figures S14 and S15). Moreover, regions are relocated only within their containing hemisphere so that arrangements were more biologically plausible.

### Age and Sex Comparison

To compare differences in network features across sexes and age groups, we conducted nonparametric permutation testing. We computed the network feature for all subjects, followed by interchanging subjects between either sex or age groups based on a random permutation, preserving the original sizes of the groups. We then compared the mean difference between the original groups with that of one million permutations, and computed the proportion of shuffled mean differences exceeding the original mean difference. The null hypothesis was rejected—there was no difference in the means between groups—at the 5% significance level if this ratio was less than 0.05.

### Component Placement Optimization

We used custom MATLAB code to conduct simulated annealing (Hastings, 1970), which identified an approximate optimal component placement of brain regions (Kaiser & Hilgetag, 2006). Each step of the simulated annealing process involves swapping the spatial position of a random pair of regions and recomputing the total wiring length. The topology of the network is maintained throughout this process. Regions are kept within their respective hemisphere to make the arrangements more biologically plausible. We repeated this search 100 times for each subject, creating 100 minimized arrangements per subject. For a given subject, the minimized arrangement used in our analyses corresponded to that with the greatest percentage reduction in wiring length across the 100 repetitions. For the maximal (worst-case) network scenario, we applied the same simulated annealing method with the goal of finding an arrangement that maximizes the total wiring length (see the Supporting Information).

### Kuramoto Model

To simulate neural activity, we used a model consisting of 82 Kuramoto oscillators (one per region). For each oscillator, the phase *θ* of oscillator *i* at time *t* + 1 is determined by the following equation:

$$\frac{d\theta_{i}}{dt}=\omega_{i}+\sum_{j=1}^{n}KA_{ij}\sin\left(\theta_{j}\left(t-\tau_{i,j}\right)-\theta_{i}\left(t\right)\right)+\eta_{i}\left(t\right),$$

where *ω*_*i*_ is the fixed natural frequency for oscillator *i* (equal to frequency in hertz multiplied by 2*π*), *K* is the global coupling strength, *A*_*ij*_ is the binary connection between oscillators *i* and *j*, *θ*_*i*_(*t*) is the phase of oscillator *i* at time *t*, and *τ*_*i*,*j*_ is the conduction delay between oscillators *i* and *j*. The matrix *A* is symmetric—communication between oscillators is bidirectional. The delay *τ*_*i*,*j*_ is defined by *τ*_*i*,*j*_ = *L*_*i*,*j*_/*V*, where *L*_*i*,*j*_ is the length of the connection between the two regions and *V* is the global conduction velocity (in meters per second). Uncorrelated Gaussian white noise *η*_*i*_(*t*) helps to ensure nonstationary dynamics, with zero mean and variance ≈2.5, 3.8, and 5.0 rad/s, for 40 Hz, 60 Hz, and 80 Hz, respectively (variance equal to 1% of natural frequency).

To calculate mean synchrony and metastability within the model, we use the instantaneous synchronization *ϕ* at time *t*, defined as (Shanahan, 2010):

$$\phi \left(t\right)=\left|{\langle e^{i\theta_{j}\left(t\right)}\rangle }_{j}\right|,$$

where *i* = $\sqrt{-1}$, *j* is an oscillator, and 〈〉_*j*_ denotes the mean over all oscillators/regions. Synchrony is calculated as the mean of *ϕ* over time, and metastability as the standard deviation of *ϕ* over time.

Selection of the appropriate *K* and *V* was obtained by comparing each subject’s simulated functional connectivity (in the original arrangement) with that of the empirical resting-state fMRI BOLD signal. For this validation process, *K* ranged from 1 to 30 (in steps of 1) and *V* from 1 to 30 m/s (in steps of 1 m/s). To enable a comparison in metastability between original and minimized arrangements, we matched the level of synchrony produced by Kuramoto models running on both arrangements (Fukushima & Sporns, 2020; Váša et al., 2015; see the Supporting Information). Subsequently, for these two separate searches, conduction velocities were reduced by 3.1 ± 1.0 m/s (reduced to 6.0 ± 2.7 m/s), 4.6 ± 1.5 m/s (reduced to 8.3 ± 3.7 m/s), and 6.1 ± 1.7 m/s (reduced to 10.8 ± 4.0 m/s), for 40 Hz, 60 Hz, and 80 Hz, respectively. For coupling strengths, these were reduced by 3.2 ± 1.4 (reduced to 5.3 ± 2.8), 5.0 ± 1.7 (reduced to 8.4 ± 3.9), and 7.0 ± 2.1(reduced to 11.1 ± 4.4). We used custom C (MEX) code within a MATLAB framework to speed up our dynamical simulations.

## SUPPORTING INFORMATION

Supporting information for this article is available at https://doi.org/10.1162/netn_a_00282.

## AUTHOR CONTRIBUTIONS

Christopher Hayward: Data curation; Investigation; Methodology; Software; Writing—Original draft. Siyu Huo: Software; Writing—Review & editing. Xue Chen: Data curation; Methodology; Software. Marcus Kaiser: Conceptualization; Funding acquisition; Methodology; Software; Supervision; Writing—Review & editing.

## FUNDING INFORMATION

Christopher James Hayward, SAgE Doctoral Training Award, Newcastle University, UK. Marcus Kaiser, Medical Research Council (https://dx.doi.org/10.13039/501100000265), Award ID: MR/T004347/1, MR/T004347/2. Marcus Kaiser, Engineering and Physical Sciences Research Council (https://dx.doi.org/10.13039/501100000266), Award ID: NS/A000026/1, EP/N031962/1, EP/W004488/1. Marcus Kaiser, Wellcome Trust, Award ID: 102037. Marcus Kaiser, Guangci Professorship Program of Ruijin Hospital, Shanghai Jiao Tong University. Xue Chen, Natural Science Foundation of Shandong Province (https://dx.doi.org/10.13039/501100007129), Award ID: ZR2018MF017. Xue Chen, China Scholarship Council, Award ID: 201706450045. Xue Chen, Fundamental Research Funds for the Central Universities, Award ID: 16CX06050A. Xue Chen, The Natural Science Foundation of Shandong Province (Grant No. ZR2019PF004).

## TECHNICAL TERMS

Connectome: The network of neural connections in the brain.
Component placement optimization (CPO): The theory that brain regions are positioned to minimize total wiring length.
Metastability: Variation in mean global synchronization over time.
BOLD fMRI: Blood oxygen level–dependent functional magnetic resonance imaging; a method of observing neural activity in the brain.

## References

[bib1] Bassett, D. S., & Bullmore, E. (2006). Small-world brain networks. The Neuroscientist, 12(6), 512–523. 10.1177/1073858406293182, 17079517

[bib2] Bullmore, E., & Sporns, O. (2009). Complex brain networks: Graph theoretical analysis of structural and functional systems. Nature Reviews Neuroscience, 10(3), 186–198. 10.1038/nrn2575, 19190637

[bib3] Bullmore, E., & Sporns, O. (2012). The economy of brain network organization. Nature Reviews Neuroscience, 13(5), 336–349. 10.1038/nrn3214, 22498897

[bib4] Cabral, J., Hugues, E., Sporns, O., & Deco, G. (2011). Role of local network oscillations in resting-state functional connectivity. NeuroImage, 57(1), 130–139. 10.1016/j.neuroimage.2011.04.010, 21511044

[bib5] Chen, B. L., Hall, D. H., & Chklovskii, D. B. (2006). Wiring optimization can relate neuronal structure and function. Proceedings of the National Academy of Sciences, 103(12), 4723–4728. 10.1073/pnas.0506806103, 16537428 PMC1550972

[bib6] Cherniak, C. (1994). Component placement optimization in the brain. Journal of Neuroscience, 14(4), 2418–2427. 10.1523/JNEUROSCI.14-04-02418.1994, 8158278 PMC6577144

[bib7] Cherniak, C., Mokhtarzada, Z., Rodriguez-Esteban, R., & Changizi, K. (2004). Global optimization of cerebral cortex layout. Proceedings of the National Academy of Sciences, 101(4), 1081–1086. 10.1073/pnas.0305212101, 14722353 PMC327154

[bib8] Cohen, J. R., & D’Esposito, M. (2016). The segregation and integration of distinct brain networks and their relationship to cognition. Journal of Neuroscience, 36(48), 12083–12094. 10.1523/JNEUROSCI.2965-15.2016, 27903719 PMC5148214

[bib9] Deco, G., Jirsa, V., McIntosh, A. R., Sporns, O., & Kötter, R. (2009). Key role of coupling, delay, and noise in resting brain fluctuations. Proceedings of the National Academy of Sciences, 106(25), 10302–10307. 10.1073/pnas.0901831106, 19497858 PMC2690605

[bib10] Deco, G., Rolls, E. T., & Romo, R. (2009). Stochastic dynamics as a principle of brain function. Progress in Neurobiology, 88(1), 1–16. 10.1016/j.pneurobio.2009.01.006, 19428958

[bib11] Desikan, R. S., Ségonne, F., Fischl, B., Quinn, B. T., Dickerson, B. C., Blacker, D., … Killiany, R. J. (2006). An automated labeling system for subdividing the human cerebral cortex on MRI scans into gyral based regions of interest. NeuroImage, 31(3), 968–980. 10.1016/j.neuroimage.2006.01.021, 16530430

[bib12] Doron, K. W., & Gazzaniga, M. S. (2008). Neuroimaging techniques offer new perspectives on callosal transfer and interhemispheric communication. Cortex, 44(8), 1023–1029. 10.1016/j.cortex.2008.03.007, 18672233

[bib13] Fischl, B., Salat, D. H., Busa, E., Albert, M., Dieterich, M., Haselgrove, C., … Dale, A. M. (2002). Whole brain segmentation: Automated labeling of neuroanatomical structures in the human brain. Neuron, 33(3), 341–355. 10.1016/S0896-6273(02)00569-X, 11832223

[bib14] Fries, P. (2015). Rhythms for cognition: Communication through coherence. Neuron, 88(1), 220–235. 10.1016/j.neuron.2015.09.034, 26447583 PMC4605134

[bib15] Friston, K. J. (1997). Transients, metastability, and neuronal dynamics. NeuroImage, 5(2), 164–171. 10.1006/nimg.1997.0259, 9345546

[bib16] Fukushima, M., & Sporns, O. (2020). Structural determinants of dynamic fluctuations between segregation and integration on the human connectome. Communications Biology, 3(1), 606. 10.1038/s42003-020-01331-3, 33097809 PMC7584581

[bib17] Gollo, L. L., Roberts, J. A., Cropley, V. L., Di Biase, M. A., Pantelis, C., Zalesky, A., & Breakspear, M. (2018). Fragility and volatility of structural hubs in the human connectome. Nature Neuroscience, 21(8), 1107–1116. 10.1038/s41593-018-0188-z, 30038275

[bib18] Hagmann, P., Cammoun, L., Gigandet, X., Meuli, R., Honey, C. J., Wedeen, V. J., & Sporns, O. (2008). Mapping the structural core of human cerebral cortex. PLoS Biology, 6(7), e159. 10.1371/journal.pbio.0060159, 18597554 PMC2443193

[bib19] Hastings, W. K. (1970). Monte Carlo sampling methods using Markov chains and their applications. Biometrika, 57(1), 97–109. 10.1093/biomet/57.1.97

[bib20] Hellyer, P. J., Scott, G., Shanahan, M., Sharp, D. J., & Leech, R. (2015). Cognitive flexibility through metastable neural dynamics is disrupted by damage to the structural connectome. Journal of Neuroscience, 35(24), 9050–9063. 10.1523/JNEUROSCI.4648-14.2015, 26085630 PMC4469735

[bib21] Hilgetag, C. C., & Kaiser, M. (2004). Clustered organization of cortical connectivity. Neuroinformatics, 2(3), 353–360. 10.1385/NI:2:3:353, 15365196

[bib22] Hill, J., Inder, T., Neil, J., Dierker, D., Harwell, J., & Van Essen, D. (2010). Similar patterns of cortical expansion during human development and evolution. Proceedings of the National Academy of Sciences, 107(29), 13135–13140. 10.1073/pnas.1001229107, 20624964 PMC2919958

[bib23] Huo, S., Zou, Y., Kaiser, M., & Liu, Z. (2022). Time-limited self-sustaining rhythms and state transitions in brain networks. Physical Review Research, 4(2), 023076. 10.1103/PhysRevResearch.4.023076

[bib24] Jung, R. E., & Haier, R. J. (2007). The Parieto-Frontal Integration Theory (P-FIT) of intelligence: Converging neuroimaging evidence. Behavioral and Brain Sciences, 30(2), 135–154. 10.1017/S0140525X07001185, 17655784

[bib25] Kaiser, M., & Hilgetag, C. C. (2006). Nonoptimal component placement, but short processing paths, due to long-distance projections in neural systems. PLoS Computational Biology, 2(7), e95. 10.1371/journal.pcbi.0020095, 16848638 PMC1513269

[bib26] Kuramoto, Y. (1975). Self-entrainment of a population of coupled non-linear oscillators. In International symposium on mathematical problems in theoretical physics (pp. 420–422). 10.1007/BFb0013365

[bib27] Muldoon, S. F., Pasqualetti, F., Gu, S., Cieslak, M., Grafton, S. T., Vettel, J. M., & Bassett, D. S. (2016). Stimulation-based control of dynamic brain networks. PLoS Computational Biology, 12(9), e1005076. 10.1371/journal.pcbi.1005076, 27611328 PMC5017638

[bib28] Park, S. H., & Lefebvre, J. (2020). Synchronization and resilience in the Kuramoto white matter network model with adaptive state-dependent delays. Journal of Mathematical Neuroscience, 10(1), 16. 10.1186/s13408-020-00091-y, 32936367 PMC7494726

[bib29] Roberts, J. A., Perry, A., Lord, A. R., Roberts, G., Mitchell, P. B., Smith, R. E., … Breakspear, M. (2016). The contribution of geometry to the human connectome. NeuroImage, 124:379–393. 10.1016/j.neuroimage.2015.09.009, 26364864

[bib30] Samu, D., Seth, A. K., & Nowotny, T. (2014). Influence of wiring cost on the large-scale architecture of human cortical connectivity. PLoS Computational Biology, 10(4), e1003557. 10.1371/journal.pcbi.1003557, 24699277 PMC3974635

[bib31] Seguin, C., van den Heuvel, M. P., & Zalesky, A. (2018). Navigation of brain networks. Proceedings of the National Academy of Sciences, 115(24), 6297–6302. 10.1073/pnas.1801351115, 29848631 PMC6004443

[bib32] Shanahan, M. (2010). Metastable chimera states in community-structured oscillator networks. Chaos: An Interdisciplinary Journal of Nonlinear Science, 20(1), 013108. 10.1063/1.3305451, 20370263

[bib33] Shen, J. P., & Lipasti, M. H. (2013). Modern processor design: Fundamentals of superscalar processors. Waveland Press, p. 20.

[bib34] Sporns, O. (2013). Network attributes for segregation and integration in the human brain. Current Opinion in Neurobiology, 23(2), 162–171. 10.1016/j.conb.2012.11.015, 23294553

[bib35] Sporns, O., Chialvo, D. R., Kaiser, M., & Hilgetag, C. C. (2004). Organization, development and function of complex brain networks. Trends in Cognitive Sciences, 8(9), 418–425. 10.1016/j.tics.2004.07.008, 15350243

[bib36] Sporns, O., Tononi, G., & Kötter, R. (2005). The human connectome: A structural description of the human brain. PLoS Computational Biology, 1(4), e42. 10.1371/journal.pcbi.0010042, 16201007 PMC1239902

[bib37] Van Essen, D. C., Ugurbil, K., Auerbach, E., Barch, D., Behrens, T. E. J., Bucholz, R., … WU-Minn HCP Consortium. (2012). The Human Connectome Project: A data acquisition perspective. NeuroImage, 62(4), 2222–2231. 10.1016/j.neuroimage.2012.02.018, 22366334 PMC3606888

[bib38] Váša, F., Shanahan, M., Hellyer, P. J., Scott, G., Cabral, J., & Leech, R. (2015). Effects of lesions on synchrony and metastability in cortical networks. NeuroImage, 118, 456–467. 10.1016/j.neuroimage.2015.05.042, 26049146

[bib39] von der Malsburg, C. (1995). Binding in models of perception and brain function. Current Opinion in Neurobiology, 5(4), 520–526. 10.1016/0959-4388(95)80014-X, 7488855

[bib40] Watts, D. J., & Strogatz, S. H. (1998). Collective dynamics of “small-world” networks. Nature, 393(6684), 440–442. 10.1038/30918, 9623998

[bib41] Waxman, S. G. (1977). Conduction in myelinated, unmyelinated, and demyelinated fibers. Archives of Neurology, 34(10), 585–589. 10.1001/archneur.1977.00500220019003, 907529

[bib42] Wildie, M., & Shanahan, M. (2012). Metastability and chimera states in modular delay and pulse-coupled oscillator networks. Chaos: An Interdisciplinary Journal of Nonlinear Science, 22(4), 043131. 10.1063/1.4766592, 23278066

