# Supplementary material for: Nonoptimal component placement of the human connectome supports variable brain dynamics
Source: Netw Neurosci. 2023 Jan 1;7(1):254–68. doi: 10.1162/netn_a_00282 (PMC10270717; doi:10.1162/netn_a_00282)
Supplement: Supplementary file 1 [file netn-7-1-254-s001.pdf]

Hayward, C. J., Huo, S., Chen, X. & Kaiser, M. (2023). Supporting information for "Non-optimal component placement of the human connectome variable brain dynamics. *Network Neuroscience, 7(1),* 254–268. https://doi.or/10.1162/netn_a_00282

Supporting Material

Non-optimal component placement of the human connectome supports variable brain dynamics

Christopher James Hayward, Siyu Huo, Xue Chen, Marcus Kaiser

Corresponding author: Marcus Kaiser (Marcus.Kaiser@nottingham.ac.uk)

Materials and Methods

Scanning parameters

For the structural and diffusion data: All Human Connectome Project imaging data (Van Essen et al., 2012) were acquired on a Siemens Skyra 3T scanner with a customized SC72 gradient insert. T1w 3D MPRAGE were acquired with TR=2400ms, TE=2.14ms, TI=1000ms, flip angle=8°, FOV=224x224, 0.7mm isotropic voxel, bandwidth=210Hz/px, iPAT=2, Acquisition time=7:40(min:sec). Diffusion weighted images were acquired with Spin-echo EPI sequences (b-values=0, 1000, 2000, 3000 s/mm^2 in approximately 90 gra-dient directions, TR=5520 ms, TE=89.5 ms, flip angle=78°, refocusing flip angle=160°, FOV=210x180 (RO x PE) matrix=168x144 (RO x PE), slice thickness=1.25mm, 111 slices, 1.25mm isotropic voxels, Multiband factor=3, Echo spacing=0.78ms, BW=1488 Hz/Px, Phase partial Fourier 6/8.

For the resting-state fMRI: Sequence: Gradient-echo PLI, TR: 720ms, TE: 33.1ms, flip angle 52 deg, FOV: 208x180 mm (RO x PE), Matrix: 104x90 (RO x PE), Slice thickness: 2.0 mm; 72 slices; 2.0 mm isotropic voxels. Multiband factor: 8, Echo spacing: 0.58 ms, BW: 2290 Hz/Px. Resting state data produced 1200 frames per run (time points), of total duration: 14 minutes 33 seconds. For the oblique axial acquisitions, we used phase encoding in a left-to-right direction. Subjects were told to keep their eyes open with relaxed fixation on a projected bright cross-hair on a dark background. We used the ICA-FIX denoised rfMRI data, with spurious correlations removed from the BOLD fMRI signal, using a combination of MELODIC and FSL FIX. For further details, see Smith et al. (2013).

Structural Data Processing and Tractography

Structural and diffusion imaging data was obtained from the Human Connectome Project (S1200 release). We used pre-processed T1-weighted 3T MRI (download: <subjectID> 3T Structural preproc extended) and pre-processed diffusion imaging data (download: <subjectID> 3T Diffusion preproc). An additional bias field correction of the diffusion images was applied using FSL fast. After applying the nodif brain mask (supplied with the diffusion imaging data) to the mean b0 image for each subject, we registered the parcellated regions from Freesurfer to the mean b0 image using an automated boundary-based registration algorithm (Freesurfer's bbregister tool).

The registered Freesurfer regions (82 in total: 34 cortical and 7 subcortical per hemisphere, based on the Desikan atlas (Desikan et al., 2006)) were used as input into DSI-Studio for deterministic fiber tracking (DSI-Studio release Jul 2019)(Yeh, Wedeen, & Tseng, 2010). Reconstruction of the diffusion images took place using generalized q-sampling imaging (GQI) reconstruction (Gangolli et al., 2017; Yeh et al., 2010) with a diffusion sampling length ratio of 1.25.

The Freesurfer aparc+aseg file was applied as a mask during the reconstruction step in DSI-Studio, ensuring that the streamlines remained within identified anatomical regions. Additionally, we used DSI-Studio to per-form two iterations of erosion during reconstructed to further prevent escaping streamlines. DSI-studio was configured to use sub-voxel seeding, initially producing 1 million streamlines per subject, with a streamline maximum turning angle of 60 degrees and a step size of 0.625. Streamlines shorter than 10mm and longer than

300mm were discarded. Streamline tracking was terminated upon contact with ventricles. After tracking, one iteration of topology-informed pruning was applied to help remove false connections (Yeh et al., 2019). All other sett ngs in DSI-Studio were set to their default values.

Output from tractography produced symmetric connectivity matrices, with entry (i, j) containing the number of streamlines with endpoints in regions i and j. For all subjects, all regions had at least one connection to another region, and that no single region was disconnected from its respective hemisphere. We did not use an arbitrary number of streamlines to threshold connections in the adjacency matrices — connections between regions were set to one if they were connected by at least one streamline, and zero otherwise. For the component placement analysis, we only considered the Euclidean distance between connected regions, independent of streamline counts. Subsequent analysis involved assessing the total reduction in wiring length when weaker connections (those with fewer streamlines) were removed.

Functional Data Processing

Resting state BOLD fMRI data was obtained from the Human Connectome Project, specifically from the S1200 resting state FIX-denoised release, consisting of 15 minutes of BOLD signal activity per subject. Using FSL fnirt, we performed a non-linear registration of each subject's structural parcellation (Freesurfer's aparc+aseg file) into the MNI space. For each region, we obtained a mask which consisted of the associated segmented cortical region and white matter (also included in the Freesurfer parcellation data). Using this combined mask, we used FSL fslmeants to extract the 1200 BOLD signal time points, repeating this for the 68 cortical and 14 sub-cortical regions across both hemispheres. Sub-cortical regions did not have an associated white matter parcellation. We applied a bandpass filter of between 0.01Hz and 0.2Hz to each region's BOLD signal. Each subject's simulated functional connectivity (FC) matrix was obtained by computing the Pearson correlation coefficient between all pairs of 82 time-series. FC matrices were used to validate our Kuramoto model of region synchronization.

Component Placement Optimization

Owing to the intractable nature of finding the component placement which minimizes the total wiring length, we used simulated annealing to approximate the minimum wiring length (Hastings, 1970). In the CPO process, for intra-hemispheric connections, we used the network consisting of straight edges between regions. The lengths of inter-hemispheric connections were lengthened as if they passed through the center-of-mass of the network, mimicking biological constraints when connecting across hemispheres: for nodes A and B (both in different hemispheres), we updated the distance of the connection between them to that of the Euclidean distance from node A to the center-of-mass of the connectome, plus the Euclidean distance from center-of-mass to node B. The center-of-mass is calculated — on a per-subject basis — as the mean spatial coordinates of all 82 regions in the connectome.

Each step of the simulated annealing process involved swapping the spatial positions of a random pair of regions and recomputing the total wiring length (the sum of the Euclidean distances between connected regions). A region's spatial position is the center-of-mass of that region (calculated from the coordinates of voxels contained in the region). During the search, the likelihood (also known as the 'temperature') of accepting a swap that increases the wiring length (accepting a less optimal state) decreases over time. The initial temperature is large enough to encourage the acceptance of less optimal states (spatial arrangements that increase the total wiring length) early on in the search. The temperature is decreased (90% of the previous iteration's temperature) after either 1000 swap attempts (independent of temperature) or 100 accepted swaps (dependent on temperature), whichever happens first. This continues until the change in the total wiring length (also known as the 'energy') falls below a predefined threshold (a maximum and minimum wiring length difference of less than 0.5%, for a particular temperature). Because only regions are moved around, the topology of the network is maintained throughout this process. To avoid local minima, we repeat this search 100 times for each subject. The spatial arrangements used in our analysis correspond to those with the greatest percentage reduction in the total wiring length obtained over these 100 repetitions, per-subject. The same parameters were used when maximizing the total wiring length, to calculate the relative wiring length. Networks were visualized using BrainNet Viewer (Xia, Wang, & He, 2013).

For volume analysis (where regions can only swap with other regions of a similar volume, according to some percentage tolerance), we calculated the volume of each region by counting the number of voxels within each region.

Kuramoto model and validation

Kuramoto oscillators (Kuramoto, 1975) coarsely approximate the patterns of excitation and inhibition observed in neural populations, a simplification of other mean field models such as the Wilson-Cowan model (Wilson & Cowan, 1973). During our simulations, each oscillator represented one of the 82 regions, with bi-directional links to other oscillators informed by the subject's binary connectome.

We validated the Kuramoto model of coupled oscillators by comparing a subject's simulated functional connectivity matrices with the functional connectivity obtained from that subject's resting state fMRI BOLD activity. By running the model for each subject, we obtained a simulated functional connectivity matrix for each pair of free parameters (coupling strength and conduction velocity). Coupling strengths ranged from 1 to 30 in steps of 1, and conduction velocities from 1 to 30m/s in steps of 1m/s. This resulted in 900 simulations, for each of the 280 subjects. The validated coupling strengths and conduction velocities were those which minimized the mean square error between the subject's simulated and empirical FC matrices. To keep the method as simple as possible, we did not alter the output of the model (such as convolving with a Haemodynamic model), with such operations showing minimal effect on the model's predictive power (Messé, Rudrauf, Benali, & Marrelec, 2014).

For each pair of free parameters (coupling strength and conduction velocity) models were simulated for 30 seconds of biological time, ignoring the first 3 seconds to allow the influence of the initial conditions to dissipate. We use Euler integration to obtain the time series of each oscillator, using a step size of 0.1 ms. In line with other studies, Kuramoto oscillators were set to have natural oscillation frequencies in the gamma range, specifically 40Hz, 60Hz and 80Hz (Váša et al., 2015). Binary matrices informed the connectivity between regions, multiplied by the coupling strength (applied equally to all connections for a given subject).

Selection of the appropriate conduction velocity and coupling strength for the minimized arrangement was as follows: Using the validated parameters for the original arrangement and the same parameter space, we conducted two separate searches over the conduction velocity and the coupling strength (each time fixing one of the empirically-validated parameters whilst changing the other). Both searches identified the single free parameter which minimized the difference in mean global synchronization (averaged over the duration of each simulation) between the original and corresponding spatially-rearranged connectome. The resulting metastability (variance in synchronization) for the minimized arrangement corresponded to the mean of the two metastability values produced by the two pairs of parameters that produce the closest match in synchrony. This approach was also used by Váša et al. (2015) and Fukushima and Sporns (2020), balancing exploration of the parameter space with exploitation of the known empirically-validated parameters for the original arrangement.

The mean synchrony for the original arrangements were 0.52±0.04 (40Hz), 0.51±0.04 (60Hz) and 0.51±0.04 (80Hz). The matched mean synchrony for the minimized arrangements were 0.54±0.05 (40Hz), 0.53±0.04 (60Hz) and 0.52±0.04 (80Hz). The corresponding metastability values for the original and minimized arrangements were: 0.13±0.04 (40Hz, original), 0.13±0.04 (60Hz, original), 0.13±0.03 (80Hz, original); 0.10±0.04 (40Hz, minimized), 0.10±0.03 (60Hz, minimized), 0.10±0.03 (80Hz, minimized).

Computing resources

This research made use of the Rocket high performance computing service at Newcastle University, UK, and the ARC4 system which is part of the high performance computing facilities at the University of Leeds, UK.

Figures and Tables

Table S1: Brain region abbreviations and full names (34 cortical and 7 subcortical regions per hemisphere)

Cortical			
BSTS	Bank SSTS	PARC	Paracentral
CAC	Caudal Anterior Cingulate	POPE	Pars Opercularis
CMF	Caudal Middle Frontal	PORB	Pars Orbitalis
CUN	Cuneus	PTRI	Pars Triangularis
ENT	Entorhinal	PCAL	Pericalcarine
FP	Frontal Pole	PSTC	Postcentral
FUS	Fusiform	PC	Posterior Cingulate
IP	Inferior Parietal	PREC	Precentral
IT	Inferior Temporal	PCUN	Precuneus
INS	Insula	RAC	Rostral Anterior Cingulate
ISTC	Isthmus Cingulate	RMF	Rostral Middle Frontal
LOCC	Lateral Occipital	SF	Superior Frontal
LOF	Lateral Orbitofrontal	SP	Superior Parietal
LING	Lingual	ST	Superior Temporal
MOF	Medial Orbitofrontal	SMAR	Supramarginal
MT	Middle Temporal	TP	Temporal Pole
PARH	Parahippocampal	TT	Transverse Temporal
Subcortical			
ACC	Nucleus Accumbens	PAL	Pallidum
AMYG	Amygdala	PUTA	Putamen
CAUD	Caudate	THAL	Thalamus
HIPP	Hippocampus		

Table S2: Bi-directional connections missing in the minimized arrangements in all subjects with that connection — for connections which were present in at least 50% of subjects prior to rearrangement.

Endpoint	Endpoint	Missing %	Eucl. wiring length
LH lateral occipital	RH lingual	100	0.85±0.02
LH lateral occipital	RH precuneus	100	0.83±0.02
RH lingual	RH frontal pole	100	0.82±0.02
LH pericalcarine	LH rostral middle frontal	100	0.79±0.02
LH cuneus	LH rostral middle frontal	100	0.79±0.02
RH cuneus	RH pars orbitalis	100	0.79±0.03
RH pericalcarine	RH rostral middle frontal	100	0.79±0.02
RH cuneus	RH rostral middle frontal	100	0.79±0.03
LH pars orbitalis	LH pericalcarine	100	0.77±0.02
RH pars orbitalis	RH pericalcarine	100	0.77±0.02
LH lateral occipital	LH superior frontal	100	0.75±0.02
RH lateral occipital	RH superior frontal	100	0.75±0.02
RH pars orbitalis	RH superior parietal	100	0.73±0.02
LH cuneus	LH lateral orbitofrontal	100	0.73±0.02
LH medial orbitofrontal	LH superior parietal	100	0.71±0.02
RH lateral orbitofrontal	RH perical carine	100	0.71±0.02
LH cuneus	LH superior frontal	100	0.67±0.02
LH cuneus	LH temporal pole	100	0.67±0.02

Note. Wiring length is normalized against the maximum wiring length for each subject. Values are mean±SD. Eucl.=Euclidean (straight-line distance). LH=left hemisphere. RH=right hemisphere.

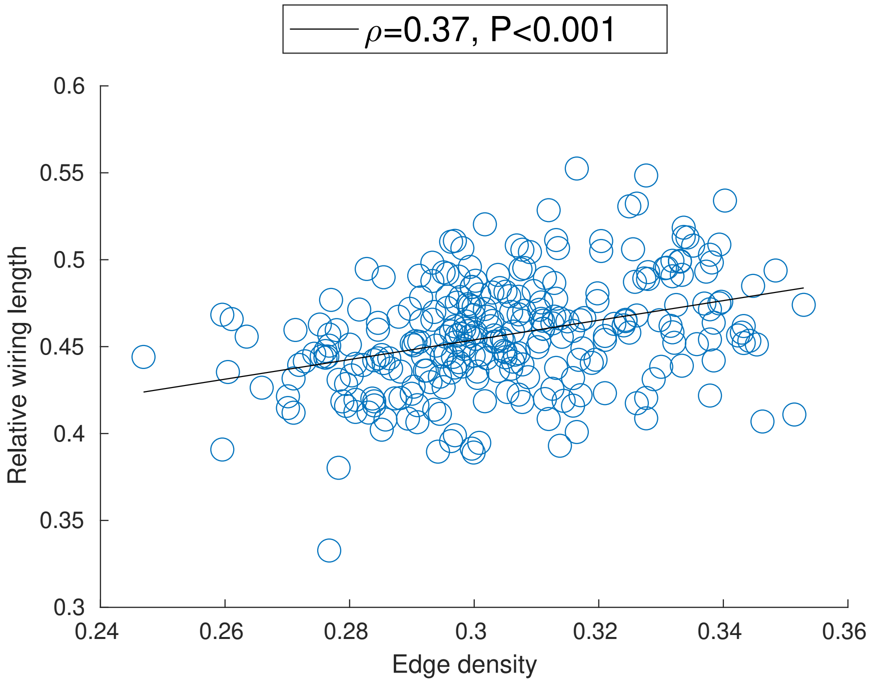

Figure S1: Comparing relative wiring length and edge density. Networks with reduced relative wiring lengths had fewer connections (ρ = Spearman rank correlation).

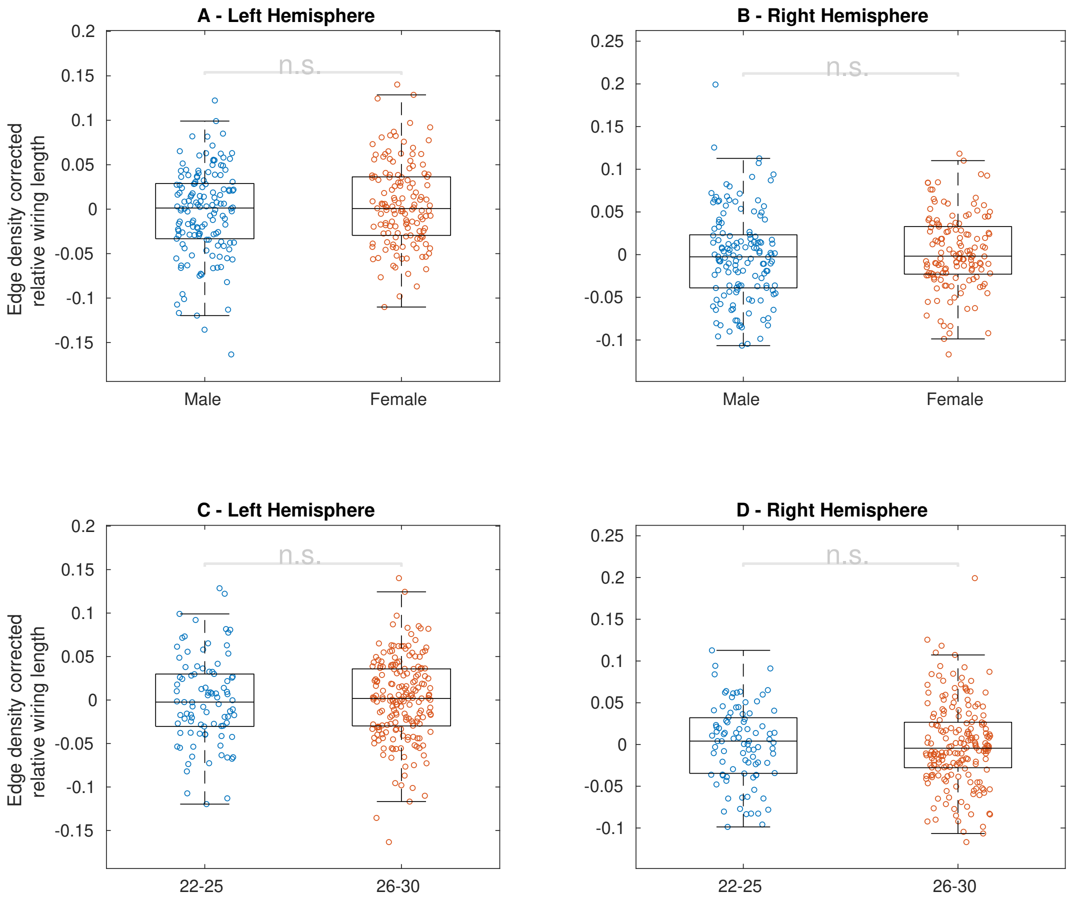

Figure S2: Comparing the corrected relative wiring lengths (adjusted for edge density) between age groups and sex. No differences were observed for **A,C:** the left hemisphere, or **B,D:** the right hemisphere (Mann-Whitney U, n.s.= $P > 0.05$).

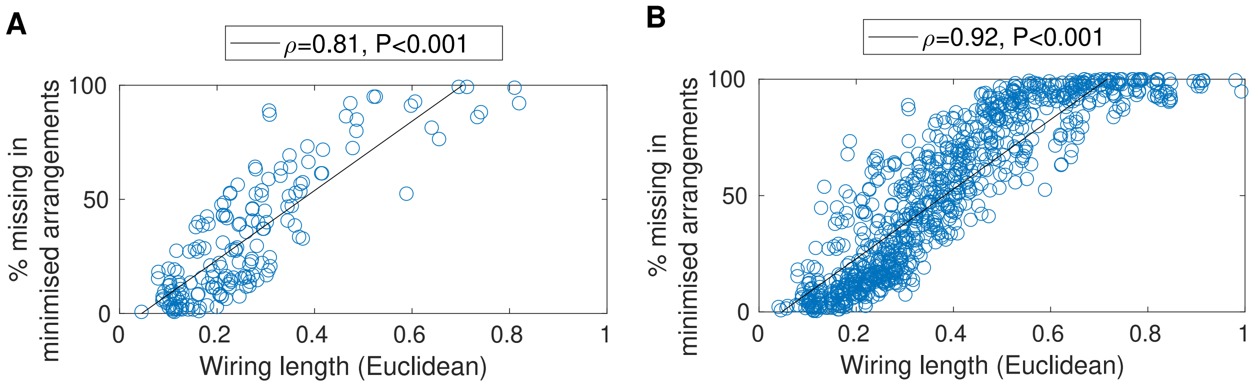

Figure S3: Connection length versus the percentage of subjects where the connection was no longer present between spatial positions in the minimized arrangements. **A:** Only considering connections which existed in all subjects prior to rearrangement (edge density = 0.05). **B:** Only considering connections which existed in at least 50% of subjects prior to rearrangement (edge density = 0.23±0.01). Longer connections were more likely to disappear in the minimized arrangements. See Table S2 for the connections which disappeared most frequently.

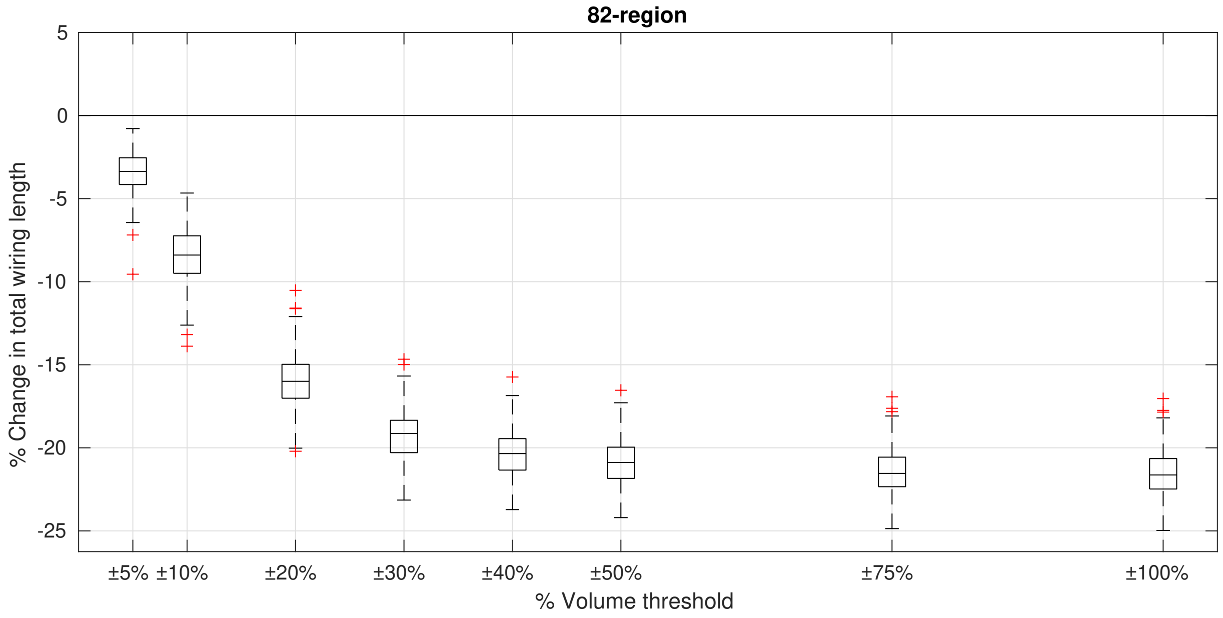

Figure S4: Assessing the effect of constraining the arrangement process based on region volume when arranging regions to minimize the total wiring length. We ran the arrangement procedure over various volume constraints (i.e. regions can only swap with other regions if they have similar volumes, defined by a percentage tolerance). As the volume constraint became less restrictive, a greater reduction in wiring length was achieved. For both parcellations, reductions in wiring length were still possible even for very restrictive constraints — with a volume restriction of ±5%, a wiring length reduction of (3.4±1.2)% was possible.

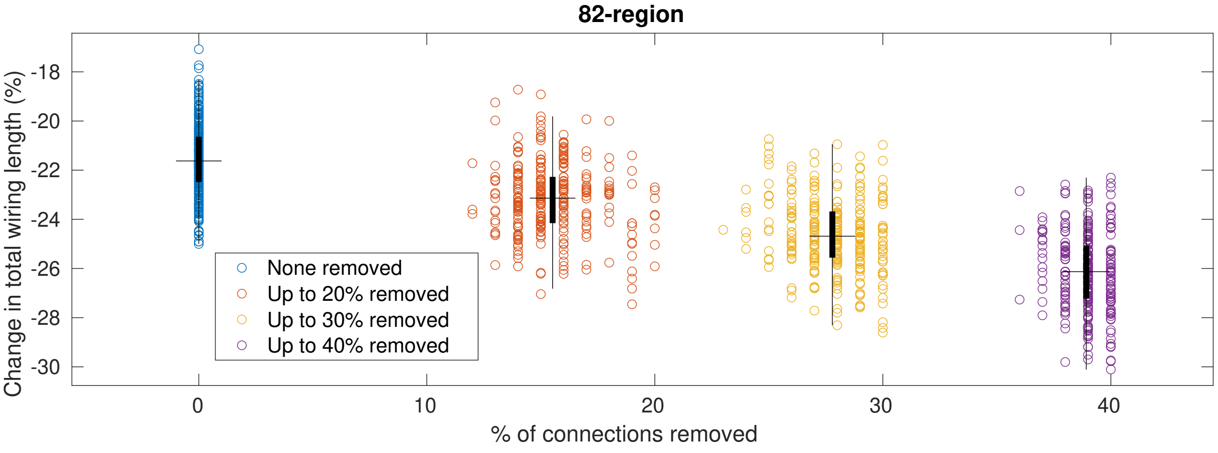

Figure S5: Performing the rearrangement procedure on connectomes with weak edges removed (those with few streamlines). Connectomes where weak edges were removed supported a greater reduction in total wiring length following rearrangement.

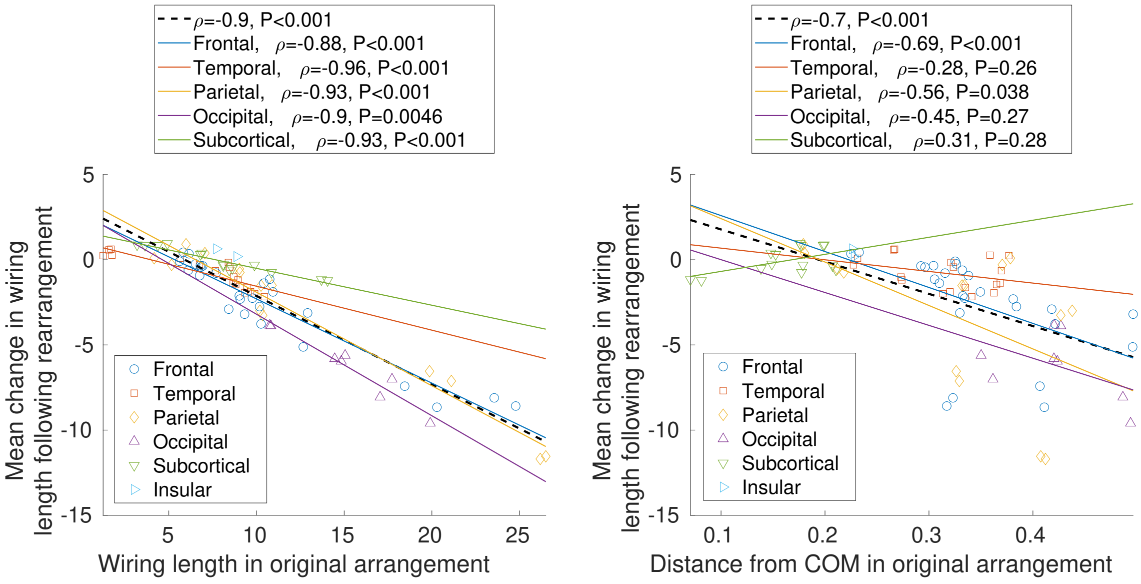

Figure S6: **Left:** Original wiring length against changes in total wiring length following rearrangement of regions to minimize the total Euclidean wiring length. Distances are normalized against the maximum Euclidean distance within each connectome. Regions with a greater reduction in total wiring length had a greater original total wiring length (ρ = Spearman rank correlation). The dashed line refers to the regression over all regions. The insular lobe was excluded from the lobe-level regression as it consists of just one region per hemisphere. **Right:** Comparing region distance from COM and change in total wiring length. Regions with a greater reduction in total wiring length were originally positioned further from the COM; nodes on the periphery of the connectome experienced a greater reduction in wiring length following rearrangement. This correlation was also significant when considering regions in the parietal lobe only.

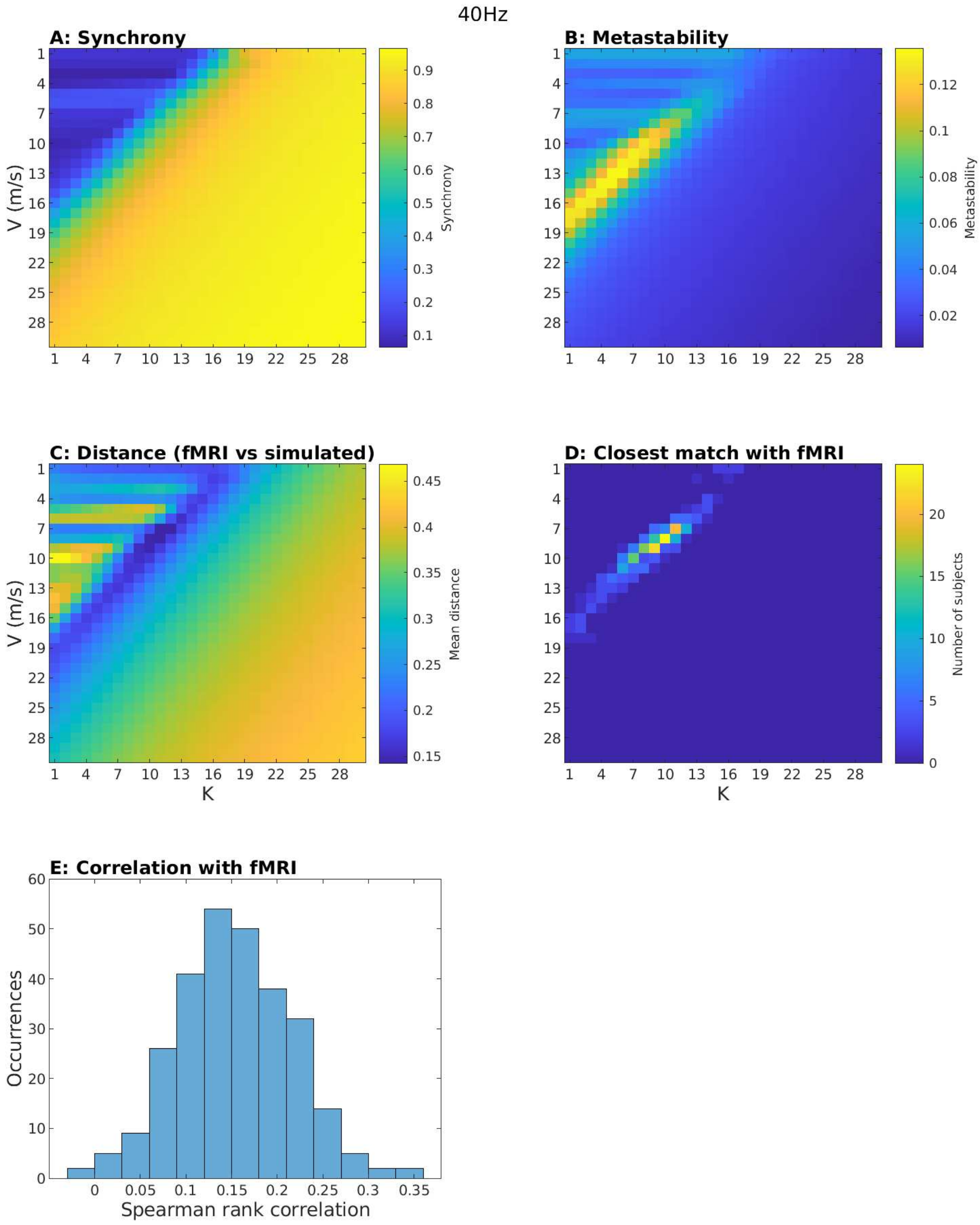

Figure S7: For an oscillation frequency of 40Hz — parameter sweep over conduction velocities and coupling strengths. For all subjects and over all conduction velocities and coupling strengths: **A:** mean synchrony; **B:** mean metastability; **C:** mean squared error between the resting-state fMRI and simulated functional connectivity matrices; **D:** the choice of V and K which minimized the mean squared error between empirical and simulated data: mean conduction velocity and coupling strength were 9.1±3.0 m/s and 8.6±3.1, respectively. At these positions, the mean values of synchrony and metastability were 0.52±0.04 and 0.13±0.04, respectively (for metastability, this equated to 61%±15 of the maximum metastability achieved over the parameter space); **E:** the Spearman correlation between empirical and simulated matrices at the point of minimum mean squared error (ρ=0.15±0.06).

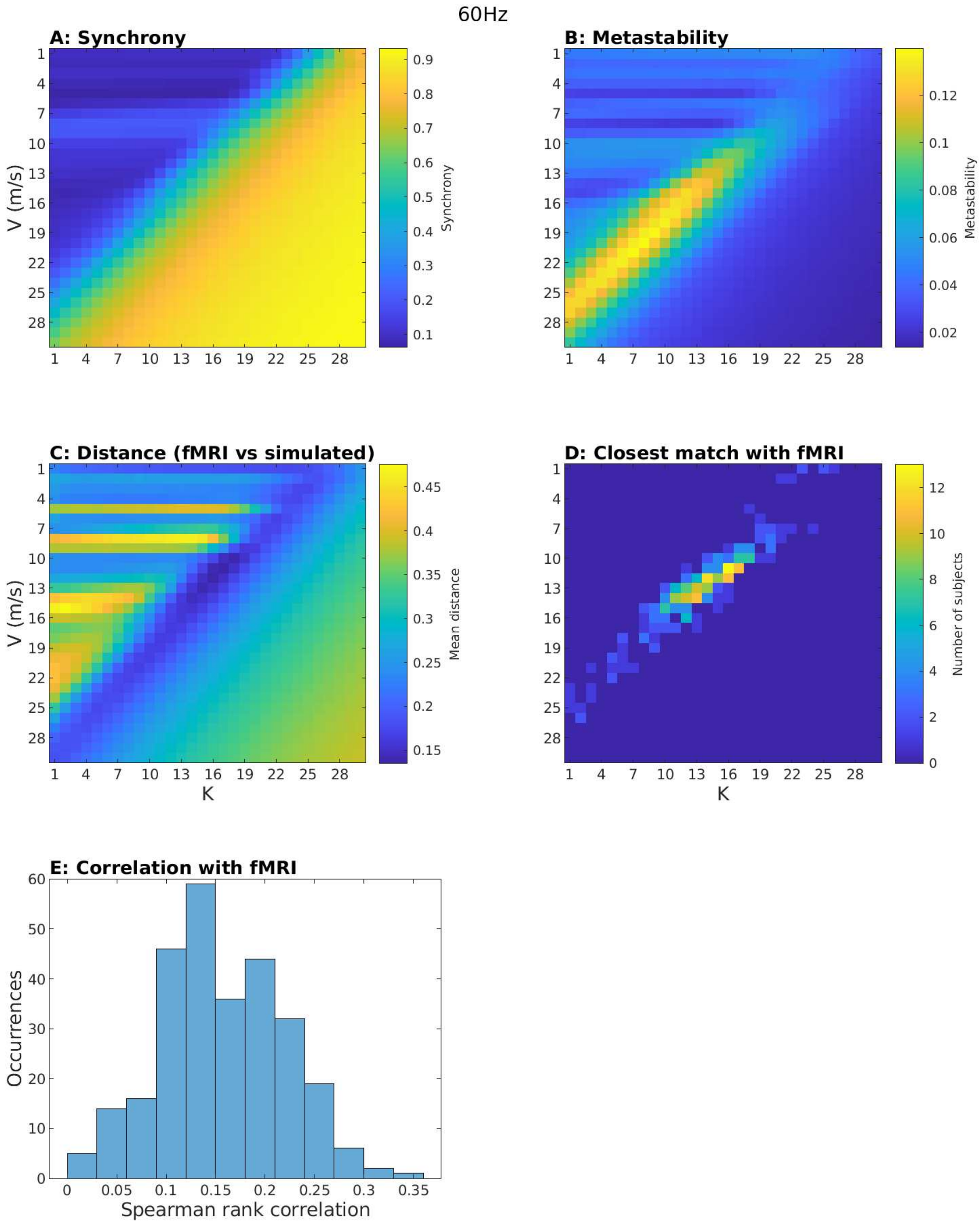

Figure S8: For an oscillation frequency of 60Hz — parameter sweep over conduction velocities and coupling strengths. For all subjects and over all conduction velocities and coupling strengths: **A:** mean synchrony; **B:** mean metastability; **C:** mean squared error between the resting-state fMRI and simulated functional connectivity matrices; **D:** the choice of V and K which minimized the mean squared error between empirical and simulated data: mean conduction velocity and coupling strength were 12.9±3.9 m/s and 13.5±4.3, respectively. At these positions, the mean values of synchrony and metastability were 0.51±0.04 and 0.13±0.04, respectively (for metastability, this equated to 59%±15 of the maximum metastability achieved over the parameter space); **E:** the Spearman correlation between empirical and simulated matrices at the point of minimum mean squared error (ρ=0.16±0.06).

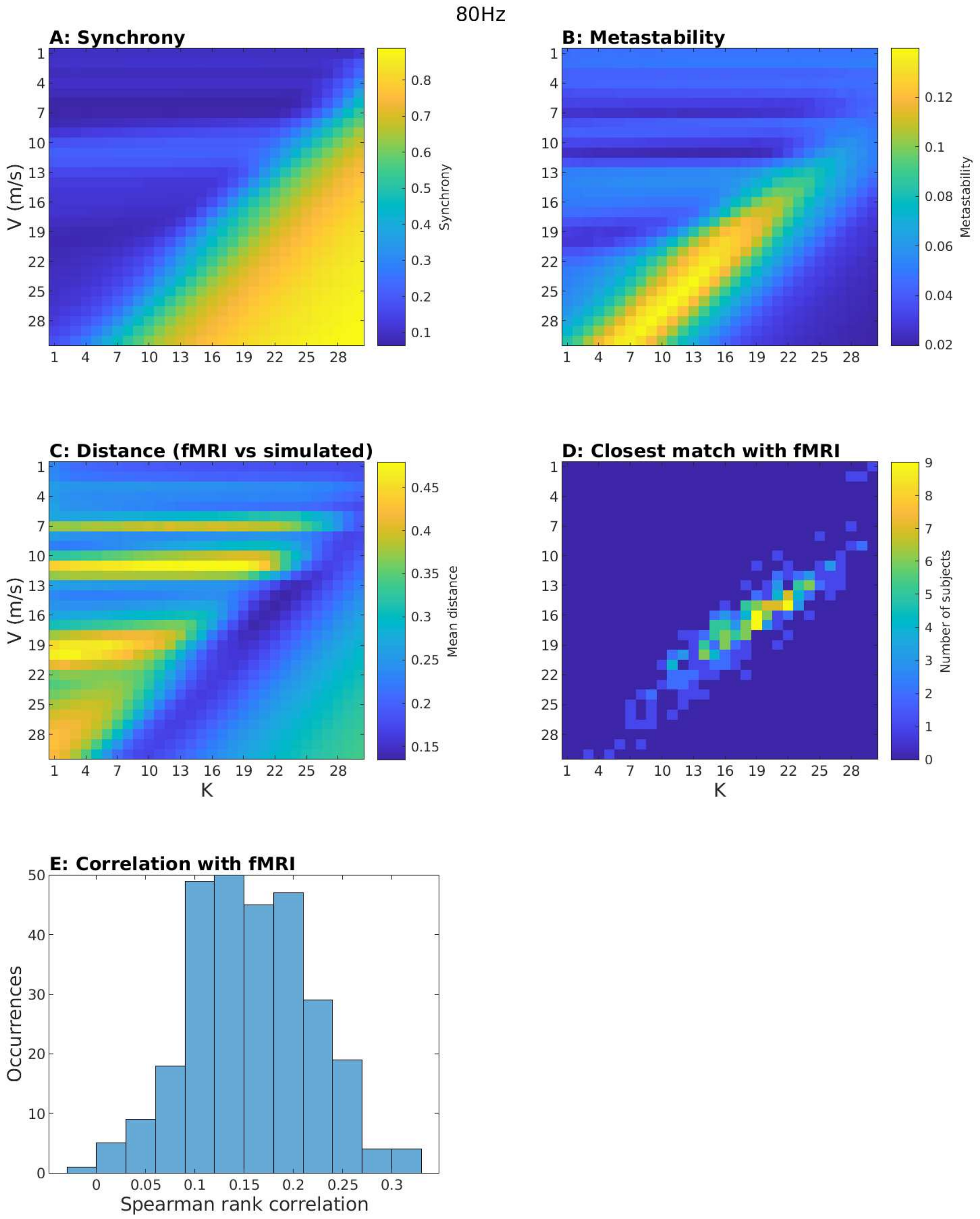

Figure S9: For an oscillation frequency of 80Hz — parameter sweep over conduction velocities and coupling strengths. For all subjects and over all conduction velocities and coupling strengths: **A:** mean synchrony; **B:** mean metastability; **C:** mean squared error between the resting-state fMRI and simulated functional connectivity matrices; **D:** the choice of V and K which minimized the mean squared error between empirical and simulated data: mean conduction velocity and coupling strength were 17.0±4.2 m/s and 18.0±4.8, respectively. At these positions, the mean values of synchrony and metastability were 0.51±0.04 and 0.13±0.03, respectively (for metastability, this equated to 59%±14 of the maximum metastability achieved over the parameter space); **E:** the Spearman correlation between empirical and simulated matrices at the point of minimum mean squared error (ρ=0.16±0.06).

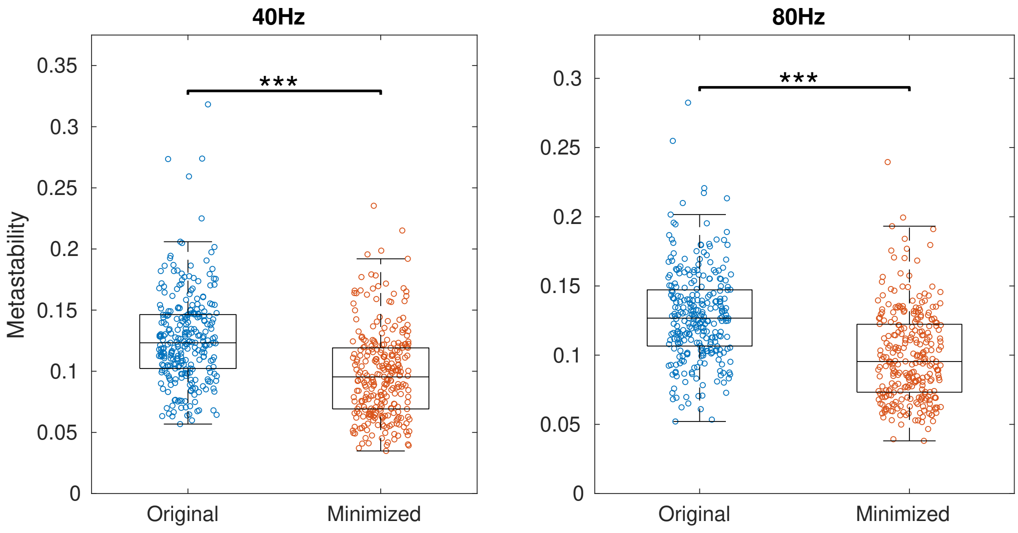

Figure S10: Change in metastability between the original and minimized spatial arrangement of brain regions. *** = $P < 0.001$, Wilcoxon signed-rank test. Cohen's $d = 0.76$ (40Hz), 0.76 (80Hz). Reductions in metastability in the minimized arrangements: 79% (40Hz), 79% (80Hz)

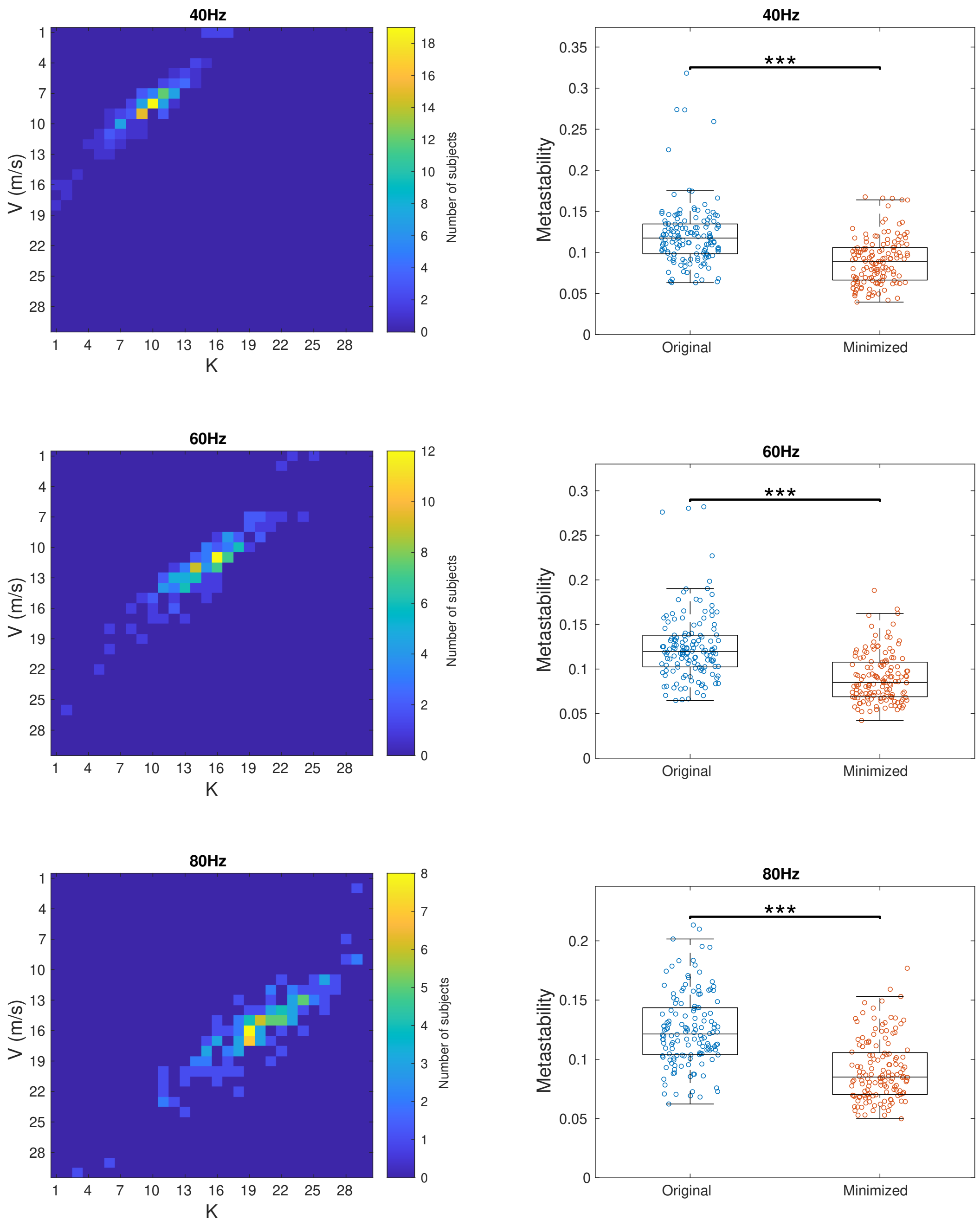

Figure S11: Changes in simulated metastability for the 50% of subjects with the lowest mean squared error between the empirical and simulated functional connectivity. **Left column:** The number of occurrences in the parameter space for which a pair of parameters minimized the distance between the fMRI and simulated functional connectivity (mean ρ with fMRI: 0.18±0.06, 0.19±0.06, 0.19±0.05, for 40, 60 and 80Hz resp.). **Right column:** The difference in metastability between the original and minimized spatial arrangements, with all frequencies showing significant reductions in metastability (*** $= P < 0.001$, Wilcoxon signed-rank test). Cohen's $d = 0.84$ (40Hz), 0.93 (60Hz), 1.02 (80Hz). Percentage of subjects showing reductions in metastability in the minimized arrangements: 81% (40Hz), 83% (60Hz), 83% (80Hz)

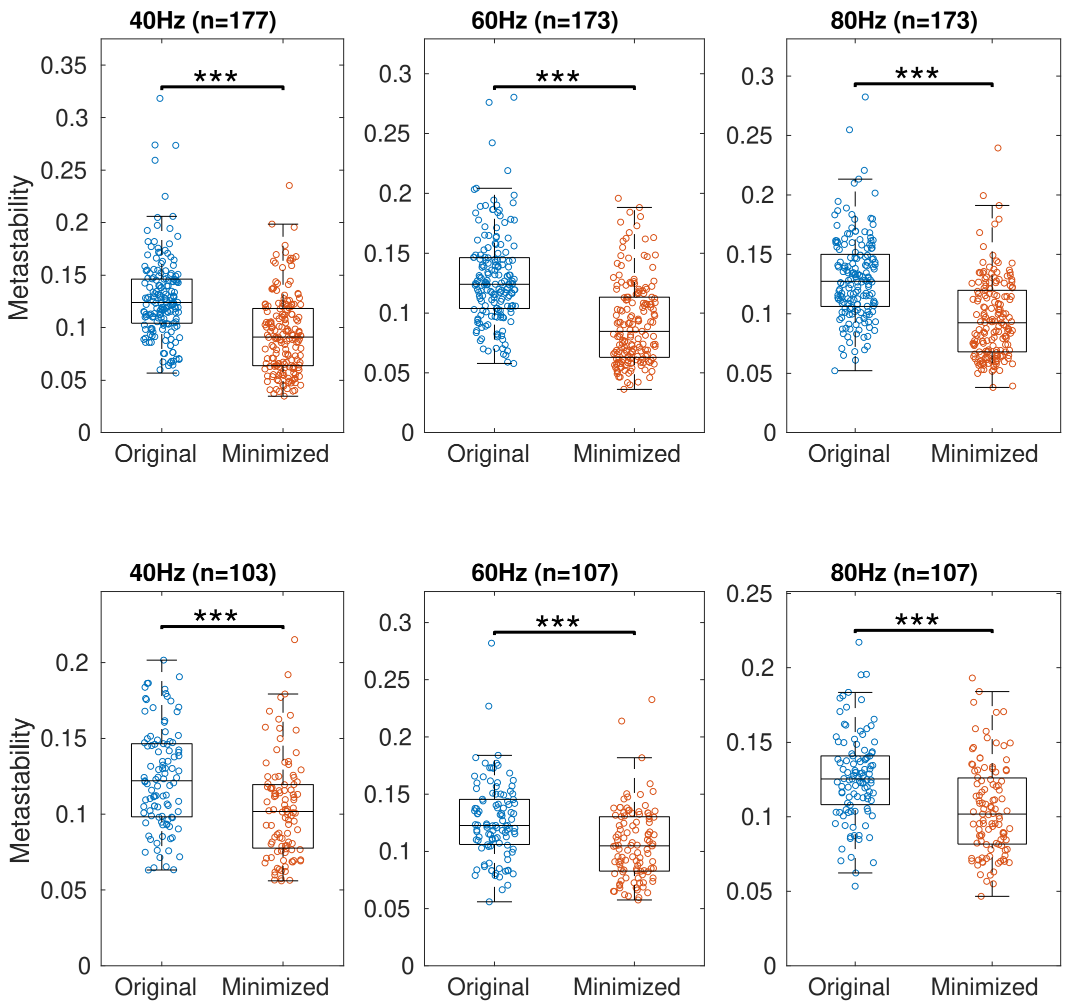

Figure S12: Changes in simulated metastability for subjects whose mean global synchrony for the minimized arrangements was above/below that of the original arrangement. **Top row:** Change in metastability for the subjects whose minimized arrangements showed greater synchrony compared with the original arrangement. Metastability was reduced in 82%, 79% and 82% of subjects, for 40Hz, 60Hz and 80Hz respectively. Cohen's d: 0.8 (40Hz), 0.9 (60Hz), 0.9 (80Hz). **Bottom row:** Change in metastability for the subjects whose minimized arrangements showed lower synchrony compared with the original arrangement. Metastability was reduced in 74%, 75% and 73% of subjects, for 40Hz, 60Hz and 80Hz respectively. Cohen's d: 0.6 (40Hz), 0.6 (60Hz), 0.7 (80Hz).

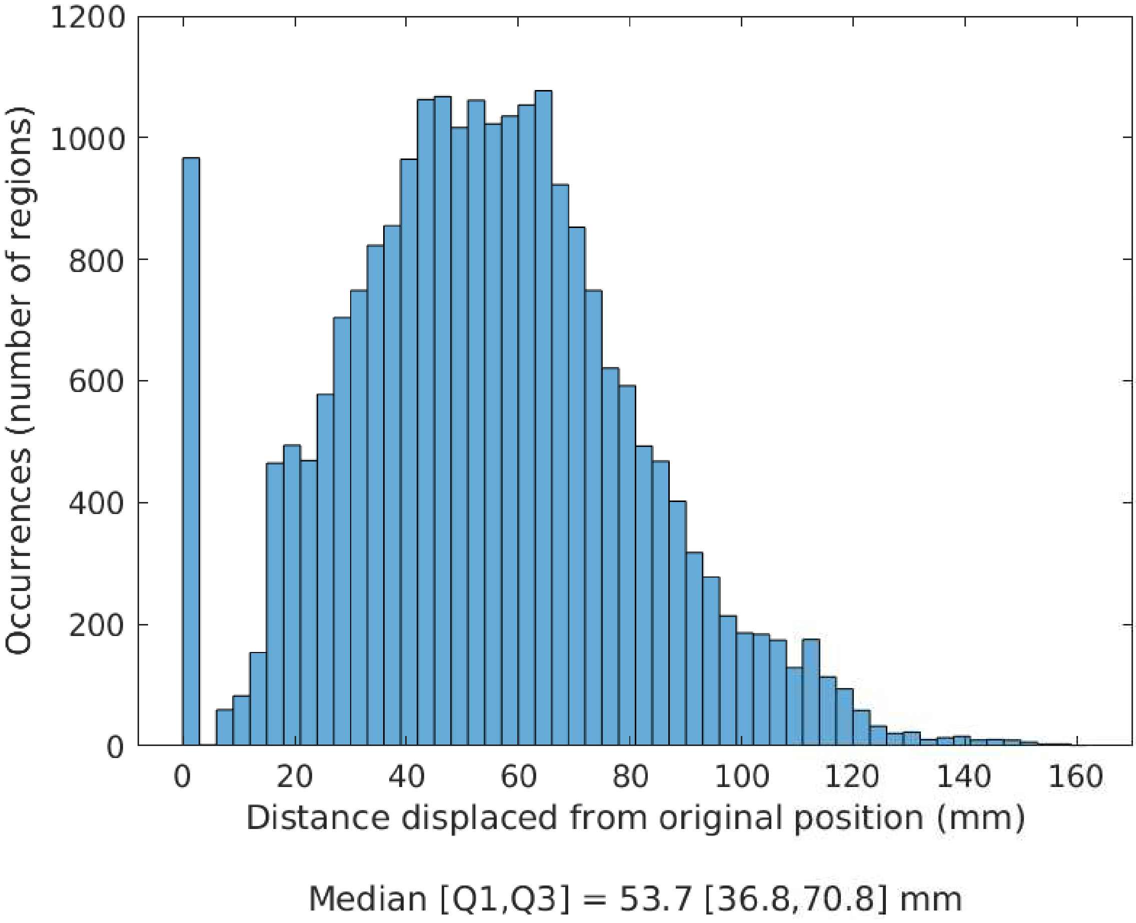

Figure S13: For the 280 subjects, the distribution of Euclidean distances moved by regions from their original position to their new position in the minimized arrangements.

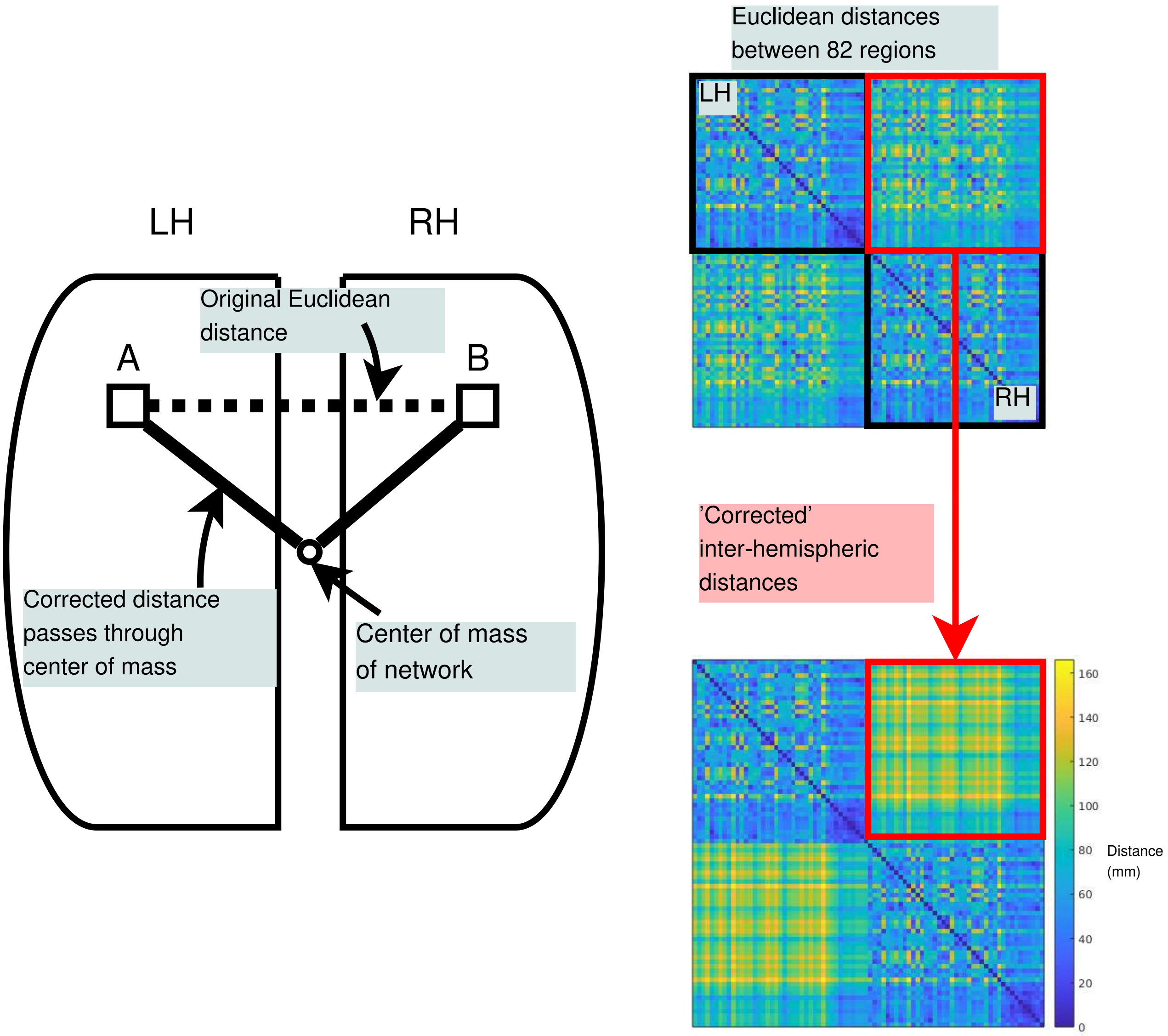

Figure S14: Visual summary of how the lengths of inter-hemispheric connections are altered as if to pass through the center-of-mass of the connectome. We change the length of inter-hemispheric connections so that the length of a connection between node A and node B is the sum of the distances from the center-of-mass of the network to A and B. Matrices containing the Euclidean distance between regions are shown on the RHS.

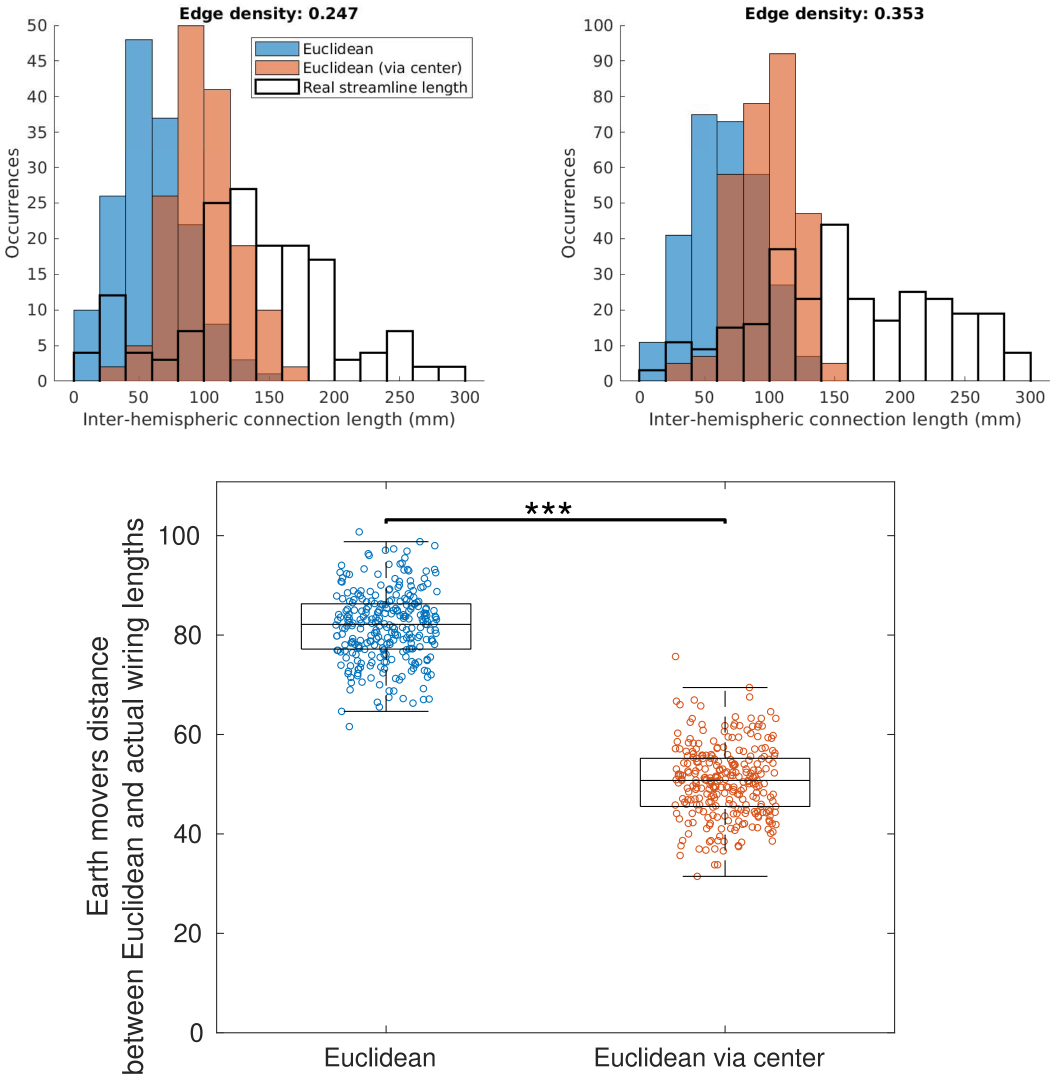

Figure S15: The effect of lengthening inter-hemispheric connections by routing through the center of mass of the connectome. **Top**: For two example connectomes — one with the minimum and another with the maximum edge density (0.247 and 0.353, resp.) from the 280 subjects — the distribution of connection lengths when connections are approximated as straight lines (Euclidean); when the lengths of inter-hemispheric connections are increased as if to pass through the center-of-mass of the connectomes (Euclidean via-center); and the real fibre tract lengths. **Bottom**: The difference in distributions between the two Euclidean approximations and the real wiring length across all 280 subjects, quantified by the earth movers distance (Rubner et al., 2000). An improved match occurs when connections are lengthened as if to pass through the center-of-mass of the connectome (*** = P<0.001, Wilcoxon signed-rank).

References

Desikan, R. S., Ségonne, F., Fischl, B., Quinn, B. T., Dickerson, B. C., Blacker, D., ... others (2006). An automated labeling system for subdividing the human cerebral cortex on mri scans into gyral based regions of interest. *Neuroimage*, *31*(3), 968–980.

Fukushima, M., & Sporns, O. (2020). Structural determinants of dynamic fluctuations between segregation and integration on the human connectome. *bioRxiv*.

Gangolli, M., Holleran, L., Kim, J. H., Stein, T. D., Alvarez, V., McKee, A. C., & Brody, D. L. (2017). Quantitative validation of a nonlinear histology-mri coregistration method using generalized q-sampling imaging in complex human cortical white matter. *Neuroimage*, *153*, 152–167.

Hastings, W. K. (1970). *Monte Carlo sampling methods using Markov chains and their applications.* Oxford University Press.

Kuramoto, Y. (1975). Self-entrainment of a population of coupled non-linear oscillators. In *International symposium on mathematical problems in theoretical physics* (pp. 420–422).

Messé, A., Rudrauf, D., Benali, H., & Marrelec, G. (2014). Relating structure and function in the human brain: relative contributions of anatomy, stationary dynamics, and non-stationarities. *PLoS computational biology*, *10*(3).

Rubner, Y., Tomasi, C., & Guibas, L. J. (2000). The earth mover's distance as a metric for image retrieval. *International Journal of Computer Vision*, *40*(2), 99–121.

Smith, S. M., Beckmann, C. F., Andersson, J., Auerbach, E. J., Bijsterbosch, J., Douaud, G., ... others (2013). Resting-state fmri in the human connectome project. *Neuroimage*, *80*, 144–168.

Van Essen, D. C., Ugurbil, K., Auerbach, E., Barch, D., Behrens, T., Bucholz, R., ... others (2012). The human connectome project: a data acquisition perspective. *Neuroimage*, *62*(4), 2222–2231.

Váša, F., Shanahan, M., Hellyer, P. J., Scott, G., Cabral, J., & Leech, R. (2015). Effects of lesions on synchrony and metastability in cortical networks. *Neuroimage*, *118*, 456–467.

Wilson, H. R., & Cowan, J. D. (1973). A mathematical theory of the functional dynamics of cortical and thalamic nervous tissue. *Kybernetik*, *13*(2), 55–80.

Xia, M., Wang, J., & He, Y. (2013). Brainnet viewer: a network visualization tool for human brain connectomics. *PloS ONE*, *8*(7), e68910.

Yeh, F.-C., Panesar, S., Barrios, J., Fernandes, D., Abhinav, K., Meola, A., & Fernandez-Miranda, J. C. (2019). Automatic removal of false connections in diffusion mri tractography using topology-informed pruning (tip). *Neurotherapeutics*, *16*(1), 52–58.

Yeh, F.-C., Wedeen, V. J., & Tseng, W.-Y. I. (2010). Generalized q-sampling imaging. *IEEE transactions on medical imaging*, *29*(9), 1626–1635.
